# Years of life lost due to cancer in the United Kingdom from 1988 to 2017

**DOI:** 10.1038/s41416-023-02422-8

**Published:** 2023-09-19

**Authors:** Amar S. Ahmad, Judith Offman, Christine Delon, Bernard V. North, Jon Shelton, Peter D. Sasieni

**Affiliations:** 1 Cancer Intelligence, Cancer Research UK, London, UK; 2 Cancer Prevention Group, School of Cancer and Pharmaceutical Sciences, King’s College London, London, UK; 3 Wolfson Institute of Population Health, Queen Mary University of London, London, UK; 4 Cancer Research UK and King’s College London Cancer Prevention Trials Unit, Cancer Prevention Group, School of Cancer and Pharmaceutical Sciences, King’s College London, London, UK

**Keywords:** Epidemiology, Cancer, Epidemiology

## Abstract

**Background:**

We investigated the application of years of life lost (YLL) in routine cancer statistics using cancer mortality data from 1988 to 2017.

**Methods:**

Cancer mortality data for 17 cancers and all cancers in the UK from 1988 to 2017 were provided by the UK Association of Cancer Registries by sex, 5-year age group, and year. YLL, age-standardised YLL rate (ASYR) and age-standardised mortality rate (ASMR) were estimated.

**Results:**

The annual average YLL due to cancer, in the time periods 1988–1992 and 2013–2017, were about 2.2 and 2.3 million years, corresponding to 4510 and 3823 ASYR per 100,000 years, respectively. During 2013–2017, the largest number of YLL occurred in lung, bowel and breast cancer. YLL by age groups for all cancers showed a peak between 60–64 and 75–79. The relative contributions to incidence, mortality, and YLL differ between cancers. For instance, pancreas (in women and men) made up a smaller proportion of incidence (3%) but bigger proportion of mortality (6 and 5%) and YLL (5 and 6%), whereas prostate cancer (26% of incidence) contributed 13% mortality and 9% YLL.

**Conclusion:**

YLL is a useful measure of the impact different cancers have on society and puts a higher weight on cancer deaths in younger individuals.

## Introduction

Cancer is the leading cause of death worldwide [1], and in the United Kingdom, there were ~275 cancer deaths per 100,000 per year on average between 2013 and 2017 [2]. National cancer statistics provide summary statistics on society’s burden of cancer using the number of cancer deaths, as well as crude and age-standardised cancer incidence and mortality rates [3]. However, some cancer sites can have more impact on younger people than on older people [4]. Even though incidence rates for all cancers combined were highest in people aged 85–89 years of age in the UK, adults aged 50–74 accounted for more than half of all new cancer cases in 2016–2018 [5]. Therefore, the number of years of life lost (YLL) is an additional useful summary statistic, which weighs the number of deaths at a given age group by its life expectancy. It essentially gives a greater weight to deaths at a younger age and a lower weight to deaths in older age [6]. Understanding the burden cancer places on society is important when planning services and interventions and can help determine where resources should be focused for possible new screening programmes, research programmes and where public health interventions can do the most [7].

YLL can be estimated using different methodological approaches [8]. Even though these methods share similar concepts, the total number of YLL estimated varies according to the technique used. The most commonly used methods are, firstly, the use of life tables, where the YLL are calculated from the number of deaths multiplied by the UK life expectancy at the age at which deaths occurs [9]. Secondly, the years of potential/premature life lost (YPLL) approach use a selected cut-off, e.g., age 65 or 75, giving more weight to deaths occurring at younger ages [8]. Thirdly, the Global Burden of Disease (GBD) approach, which calculates YLLs using a complex modelling approach, where a standard age-specific GBD life expectancy is applied to modelled mortality [10]. Two studies have so far estimated the YLL to cancer in the UK. Firstly, the GBD published a global study on cancer incidence, mortality and YLL from 2010 to 2019 in 2021, however, this study uses modelled mortality data for the UK and not mortality data based on death certificates collated by cancer registries. Burnet and colleagues estimated YLL for 17 cancers using the life table method, however, only on data from the East Anglian Cancer Registry for the 5-year time period 1990–1994 [11].

The overall aim of this paper is to investigate the application of YLL as an indicator of cancer death and whether YLL should be used in standard cancer research reports and publications, as are, for example, produced by Cancer Research UK or cancer registries. We have used population cancer mortality data obtained from cancer registries to estimate YLL, age-standardised YLL rate (ASYR), average YLL per cancer case and age-standardised mortality rate (ASMR) for 17 different cancers, and all cancers (C00-C97) combined in the UK for the period 1988–2017. We analysed data covering 30 years allowing an in depth discussion of how intervention strategies and new treatments impacted YLL. We chose to use the life table methodology, where we applied the residual life expectancy at each age, because it does not use a fixed upper age of death and therefore allows comparisons of YLL for different age groups. These 17 cancers were chosen as they were the most common cancers in the UK at the start of the study, and Cancer Research UK presented their standard cancer statistics routinely about these cancers.

## Methods

### Data sources

Cancer mortality data for 17 cancers (individual sites or localisation groups, defined by ICD-10 codes), and all cancers combined (C00-C97) in terms of the number of deaths in the UK (England, Scotland, Wales and Northern Ireland) for 1988–2017 were provided by the Office for National Statistics, the Information Service Division (ISD) Scotland and the Northern Ireland Cancer Registry by sex, 5-year age group (0–4, 5–9, 10–14,…, 85–89, 90+), and year of death. Life expectancy data and population data were downloaded from the Office for National Statistics by single year of age [12]. The UK life tables are calculated based on population estimates and births and deaths for a period of three consecutive years and go up to age 100 [13].

The life expectancy data was averaged to form average life expectancy for 5-year age groups, and population data was summed to form 5-year age groups. The average life expectancy was calculated for six non-overlapping (i.e., not rolling) 5-year periods: from 1988–1992 to 2013–2017. Cancer mortality data and population data were calculated and included in the statistical analysis for these six 5-year periods.

### Statistical analysis

All statistical analyses were based on the annual average of the 5-year period data (e.g., 1988–1992) for all persons. However, for C50 breast, C53 cervix, C56-C57.4 ovary, and C54-C55 uterus female cancer mortality and population data were used; and for C61 prostate male cancer mortality and population data were used.

The number of ${YLL}$ caused by a cancer site $C$, in a population of sex $S$, age $A$ and period $P$, was computed as described by The Global Health Observatory [9]

$${YL}{L}_{C,S,A,P}={N}_{C,S,A,P}\times L{E}_{S,A,P}$$

where ${N}_{C,S,A,P}$ is the number of deaths due to a cancer site $C$, in a population of sex $S$, age $A$ and period $P$, $\times$ is the multiplication symbol; and $L{E}_{S,A,P}$ is the expected life expectancy for sex $S$, age $A$ and period $P$.

Furthermore, an ASYR due to cancer site $C$, in a population of sex $S$, age $A$ and period $P$, was calculated as the following [14]

$${{ASYR}}_{C,S,A,P}=\mathop{\sum}\limits_{A}\left(\frac{{{YLL}}_{C,S,A,P}}{{{Pop}}_{S,A,P}}\times 100,000\right)\times W(A)$$

where the Greek capital sigma ($\sum$) stands for “sum”, ${{CYR}}_{C,S,A,P}$ is the crude YLL rate due to a cancer site *C*, sex *S* and age *A*, and period *P*, which was estimated as

${{Pop}}_{S,A,P}$ is the population size at sex *S* and age *A*, and period *P*; and $W(A)$ is the standard population weight at age *A* [6].

The crude mortality rate was computed similarly by dividing the cancer deaths occurring during a given time period by the size of the population among which the deaths occurred multiplied by 100,000 persons. The ASMR was computed as the weighted average of the age-specific mortality rates per 100,000 persons [15] using the European 2013 reference population [16]. The percentage change of ASYR and ASMR between the two periods 1988–1992 and 2013–2017 were calculated for 17 cancer sites. The average years of life lost (AYLL) per death for that cause was computed by dividing total YLL by the total number of deaths for each cancer site [17].

**Fig. 1:**
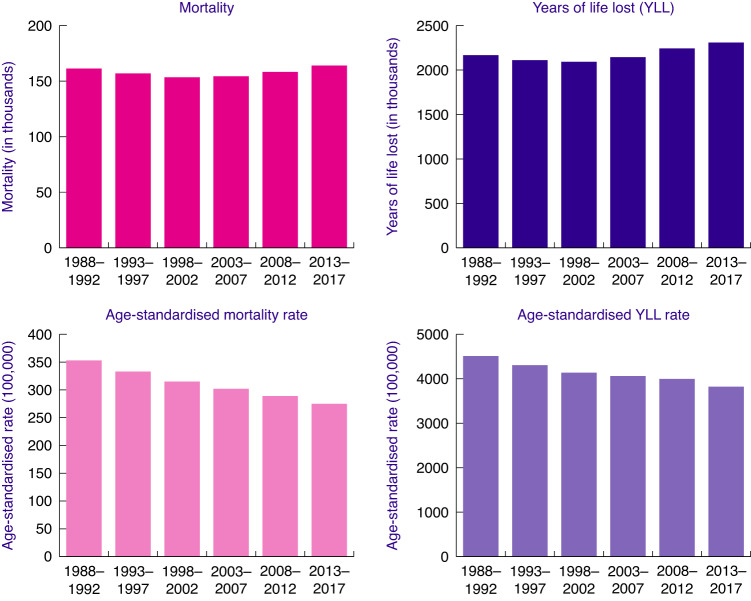
Number of all cancer (C00-97) mortality, years of life lost (YLL), age-standardised mortality rate (ASMR) and age-standardised YLL rate **(**ASYR). Statistically significant reductions were observed for both ASMR and ASYR with annual changes of −0.0493 (95% −0.0524 to −0.0462) and -0.3060 (95% CI −0.0360 to −0.0253) between the six time periods, respectively.

The annual change in ASYR and ASMR over time between the six time periods (using data in Fig. 1 and Table 1) was estimated by performing a generalised linear model with quasi-Poisson link function [18] and ASYR and ASMR as outcome variables. The independent variable was the period variable assuming linearity between the period and observed rates, which is the same as considering a constant change in the rates overtime periods. This is a major assumption, but it is generally accepted for this type of data analysis.

**Table 1:** The number of YLL and total observed number of cancer deaths (N_C_) for 17 cancer sites and all cancers combined (C00-C97).

	YLL						Death					
	1988–1992	1993–1997	1998–2002	2003–2007	2008–2012	2013–2017	1988–1992	1993–1997	1998–2002	2003–2007	2008–2012	2013–2017
C00-C97: all cancer	2,167,348	2,110,708	2,091691	2,145,020	2,242,740	2,308,277	161,296	156,965	153,434	154,302	158,266	163,979
C33-C34: lung	519,925	479,068	456,565	466,144	497,918	500,609	39,342	36,360	33,963	33,747	35,156	35,485
C18-C20: bowel	230,208	212,514	199,697	201,196	207,848	213,535	19,198	17,622	16,241	15,821	15,836	16,133
C50: breast	260,074	240,825	222,809	213,262	205,203	196,885	15,411	14,113	13,034	12,423	11,794	11,435
C25: pancreas	85,394	82,706	88,174	99,064	114,770	126,851	6815	6530	6742	7282	8142	8969
C15: oesophagus	75,634	85,502	92,979	102,298	110,931	114,249	5912	6620	6991	7354	7617	7861
C61: prostate	75,127	80,360	82,776	89,918	96,645	103,727	8956	9622	9609	10,131	10,582	11,459
C22: liver	23,741	28,759	32,970	41,921	57,635	75,613	1669	2070	2349	2885	3887	5234
C56-C57.4: ovary	75,718	75,445	75,838	73,192	70,580	68,452	4369	4413	4525	4380	4220	4124
C91-C95: leukaemia	67,730	65,233	67,178	66,575	67,493	66,947	3993	3913	4103	4313	4546	4676
C82-C86: non-Hodgkin lymphoma	61,637	67,861	69,179	65,811	64,071	65,776	3996	4343	4569	4503	4529	4830
C64-C66,C68: kidney	42,212	44,711	47,038	53,778	59,416	63,520	2807	2968	3183	3627	4028	4436
C16: Stomach	115,659	95,010	81,654	70,484	64,395	60,777	9868	8030	6741	5615	4951	4476
C67: bladder	58,198	55,285	51,982	51,681	54,731	57,357	5450	5276	4935	4846	5051	5357
C43: melanoma skin cancer	24,487	28,643	30,820	34,627	38,945	40,120	1247	1520	1647	1851	2142	2364
C90: myeloma	28,860	29,591	30,432	32,446	33,795	36,680	2389	2413	2460	2591	2655	2963
C54-C55: uterus	21,204	18,987	20,173	23,024	27,829	33,037	1605	1437	1491	1636	1874	2227
C53: cervix	43,617	33,520	27,038	23,316	22,837	21,798	1986	1526	1229	1027	949	866

Cancer sites are ranked by the average estimated number of YLL in 2013–2017.

Statistical analyses were performed in R version 4.1.0 [19].

## Results

Table 1 illustrates the estimated annual average YLL and the total observed number of cancer deaths for 17 cancer sites and for all cancers combined (C00-C97) from 1988 to 2017. The cancer sites are sorted by the estimated annual average YLL in 2013–2017. In 2013–2017, six cancer types each caused over 100,000 YLL. The annual average YLL due to lung cancer is 500,609 years, bowel cancer—213,535 years, breast cancer— 195,636 years, pancreatic—126,851 years, oesophageal—114,249 years, and prostate—103,727 years. Supplementary Table 1 furthermore shows average YLL and observed cancer deaths by sex for all cancers and for 51 different cancer sites. For men, the highest average YLL was due to lung cancer—256,985 years, bowel—114,555 years and prostrate—103,732 years, whereas for women, the highest YLL were due to lung, breast and bowel cancer, with 240,750, 195,667 and 97,151 years, respectively. In 2013–2017, the total number of YLL from all cancers combined (C00-C97) was 2,308,277 years (Table 1). The observed total number of YLL is higher in pancreatic and oesophageal cancer than in prostate cancer, although the total number of deaths is higher in prostate cancer. However, this difference seen between YLL and number of deaths is not observed when comparing age-standardised rates. The ASYR (per 100,000 persons) and the ASMR (per 100,000 persons) for 17 cancer sites and all cancers (C00-C97) are shown in Table 2. In addition, Supplementary Table 2 presents ASYR and ASMR by sex for all cancers and a total of 51 cancer sites. The observed ASYR (per 100,000 persons), in the period 2013–2017, was for lung—837 years, breast—612 years, prostate—396 years, bowel—354 years, ovary—216 years and pancreas—211 years, respectively. The estimated ASYR (per 100,000) for all cancers combined (C00-C97) for the time period 2013–2017 was 3823 for all individuals, 4084 for men and 3601 for women (Table 2). Over two million YLL were observed for all cancers combined (C00-C97) in each of the time periods 1988–1992, to 2013–2017 (Fig. 1). The number of YLL decreased for the first two time periods before increasing to its highest level of 2.3 million years in 2013–2017 (Fig. 1) and the same trend was seen in YLL (results not shown). The ASYR and the ASMR for all cancers showed significant steady decreases over this time period (Fig. 1 and Supplementary Table 3). Over the entire time period, the ASYRs and ASMRs decreased significantly for the majority of cancers, with the biggest annual decreases occurring for cervix, stomach and breast. The ASYRs and ASMRs for pancreas, oesophagus, liver and melanoma skin cancers, on the other hand, increased significantly.

**Table 2:** Age-standardised YLL rates (ASYR) and age-standardised mortality rates (ASMR), per 100,000, for 17 cancer sites and for all cancers combined (C00-C97).

	ASYR						ASMR					
	1988–1992	1993–1997	1998–2002	2003–2007	2008–2012	2013–2017	1988–1992	1993–1997	1998–2002	2003–2007	2008–2012	2013–2017
C00-C97: all cancer	4510	4306	4137	4061	3997	3823	353	333	315	302	289	275
C33-C34: lung	1095	995	922	899	901	837	84	77	70	66	64	60
C50: breast	1004	903	803	737	675	612	58	52	46	42	38	34
C61: prostate	418	430	420	426	414	396	58	59	56	54	50	48
C18-C20: bowel	489	442	401	386	373	354	43	38	34	31	29	27
C56-C57.4: ovary	295	289	281	261	238	216	17	17	16	15	14	13
C25: pancreas	181	172	177	190	206	211	15	14	14	14	15	15
C15: oesophagus	160	177	186	195	198	190	13	14	14	14	14	13
C22: liver	49	58.6	65	80	103	126	4	4	5	6	7	9
C91-C95: leukaemia	128	123	125	121	118	109	9	8	8	8	8	8
C82-C86: non-Hodgkin lymphoma	124	134	134	123	114	109	9	9	9	9	8	8
C64-C66,C68: kidney	87	91	93	102	106	105	6	6	7	7	7	7
C54-C55: uterus	81	72	75	82	94	104	6	5	5	6	6	7
C16: stomach	245	197	164	135	116	101	22	17	14	11	9	8
C67: bladder	125	116	106	101	100	96	12	11	10	10	9	9
C53: cervix	163	121	94	79	74	68	7	6	4	4	3	3
C43: melanoma skin cancer	48	55	58	62	67	66	3	3	3	4	4	4
C90: myeloma	61	62	62	63	61	61	5	5	5	5	5	5

Cancer sites are sorted by the ASYR in the period 2013–2017.

**Fig. 2:**
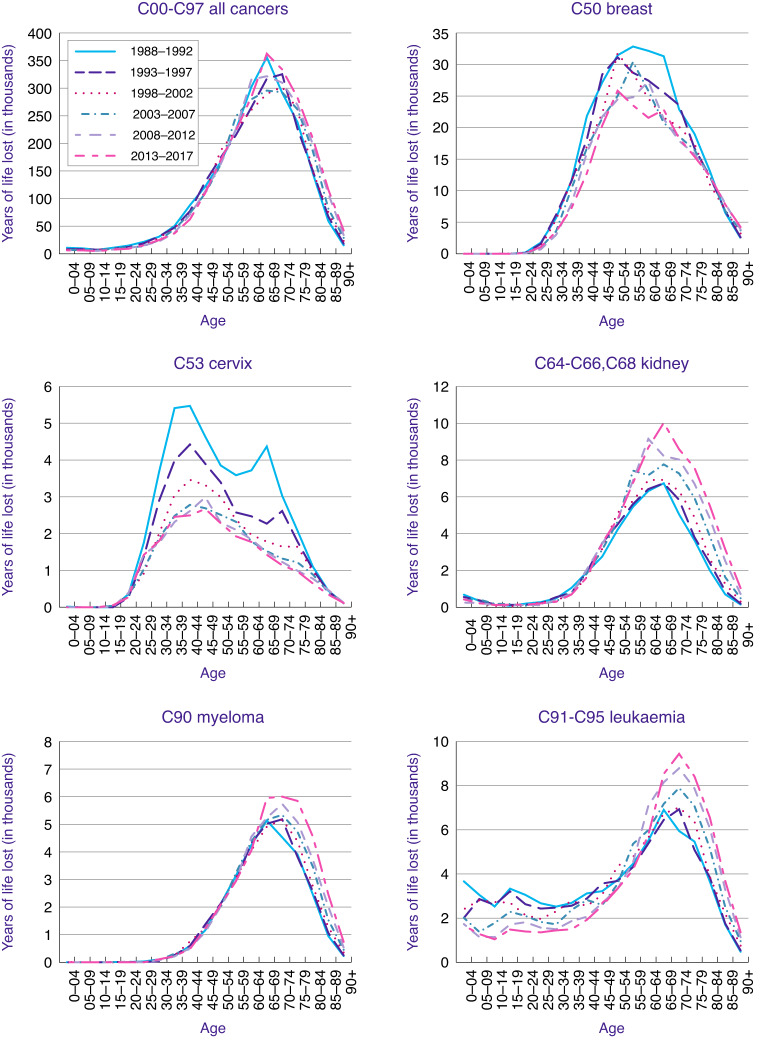
YLL by age (at death) group from 1988–1992 to 2013–2017. Graphs for all cancers, breast cancer, cervical cancer, kidney cancer, myeloma, leukaemia are included.

Figure 2 is a plot of age groups versus YLL for all cancers and six cancer sites between the time periods 1988–1992 and 2013–2017, where differences in age distribution of YLL are shown. Graphs for the other 10 cancers can be found in Supplementary Fig. 1. The number of YLL from all cancers (Fig. 2) are higher in the older age groups, between 60–64 and 75–79. Similar patterns were observed for leukaemia, myeloma, and kidney. However, for leukaemia the number of YLL are also higher in the age group <50 years as compared to other cancer sites. For breast cancer on the other hand, the number of YLL increased already from age 40, however, the number of YLL fell over the whole time period with clear reductions between 1988–1992 and 2013–2017. Figure 2 shows the number of YLL from cervical cancer, where the number of YLL fell over this time period with greater reductions in the older age groups (65–79). Only the 90+ and the under 30 age group showed no reduction in number of deaths. Furthermore, cervical cancer has the highest number of cases in younger age groups (over 60% of cancer cases occurring before age 69 compared to less than 40% for all other cancer types described here)).

**Fig. 3:**
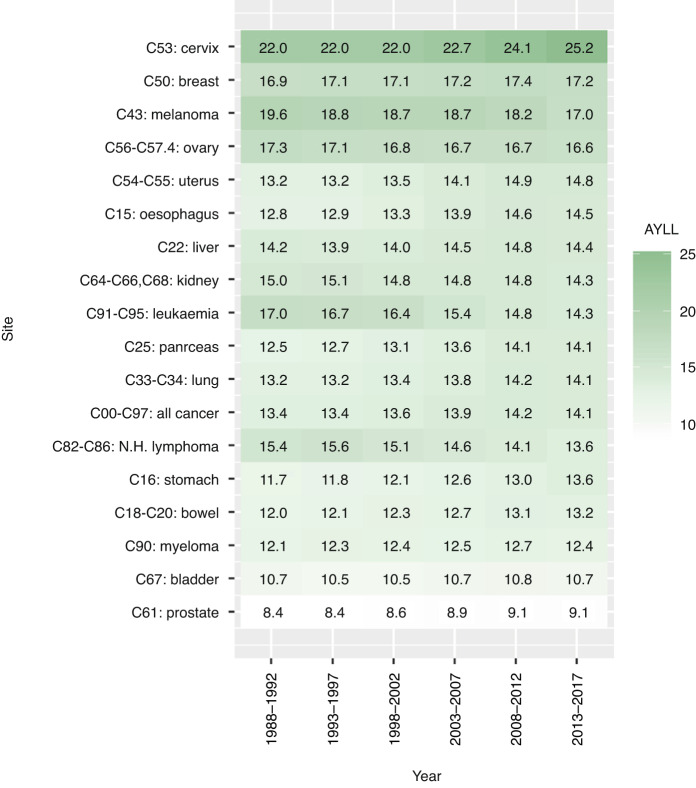
Average YLL of cancer per death for these causes estimated for six time periods. The average YLL was computed by dividing the total YLL by the total mortality, e.g., the average YLL from the lung cancer (C33-C34) at year 2013–2017 was computed as the following: 500608.86/35485 = 14.10762 (see Table 1).

Figure 3 shows AYLL of cancer estimated per death over the six time periods. In year 2013–2017, cancers of the cervix, breast, melanoma skin cancer, and ovary were most affected with AYLL 25.2, 17.2, 17.0, and 16.6 years, respectively, while prostate, bladder, myeloma, bowel, stomach cancer and non-Hodgkin lymphoma had AYLL of 9.1, 10.7, 12.4, 13.2, 13.6 and 13.6 years correspondingly. All remaining AYLL of >14 years. Estimating AYLL for men and women separately (Supplementary Table 4) identified the highest AYLL for testicular cancer in men (33 years) and cervical cancer in women (22–25 years) in addition to breast cancer in women (17 years) and melanoma skin cancer in both genders (16–20 years).

**Fig. 4:**
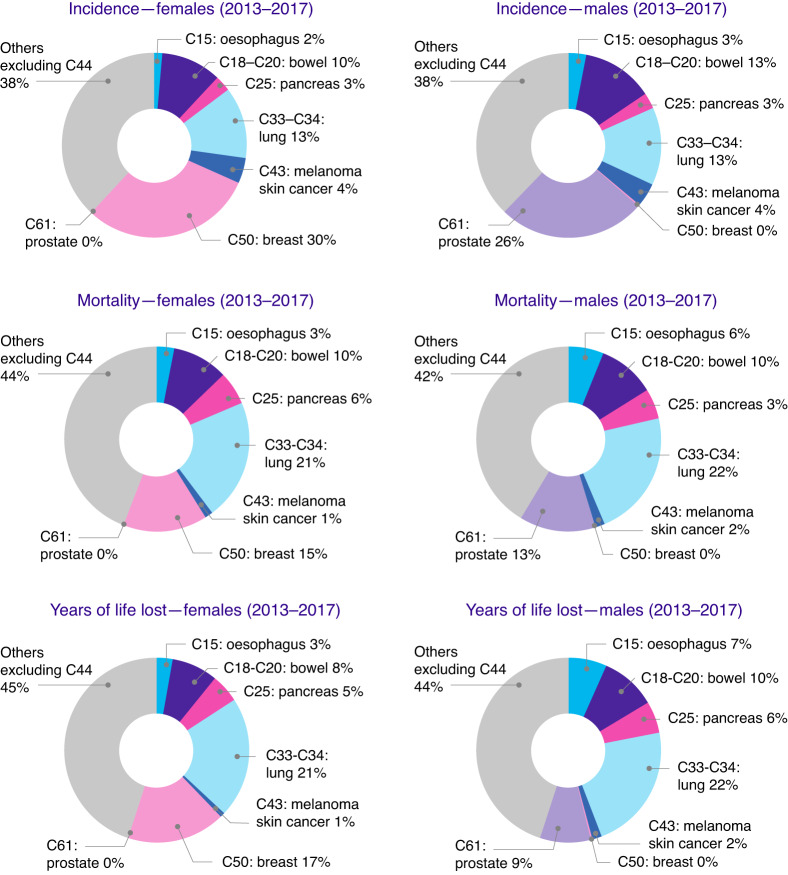
Pie charts of cancer incidence, mortality and years of life lost for females and males for 2013–2017. Only the seven most common cancers (bowel, breast, lung, melanoma skin cancer, oesophagus, pancreas, prostate) are included, and the remaining cancers are combined to ‘Others excluding C44’.

Supplementary Fig. 2 shows that, in general, there is good agreement in direction and magnitude of change in the annual average percentage change between YLL and deaths and between ASRM and ASYR, though with some exceptions. These differ primarily for prostate, bladder, non-Hodgkin lymphoma, myeloma and leukaemia, which are all cancer types that have shifted into older age groups and/or increased over time. The contributions of the most common cancers, lung, bowel, breast, prostate, oesophagus, pancreas and melanoma skin cancer to incidence, mortality and YLL are illustrated in Fig. 4. This shows that some cancers, for example, pancreas in women and men, make up a smaller proportion of cancer incidence (3%) but bigger proportion of mortality (6 and 5%) and YLL (5 and 6%). For other cancers, a large proportion of the incidence, for example, 26% for prostate cancer, results in a lower proportion of mortality (13%) and even lower YLL (9%).

## Discussion

### Summary of findings

This study reported two measures of mortality and YLL for 17 cancer sites from 1988 to 2017 based on population data obtained directly from UK cancer registries. On average, over 2 million years of life were lost to all cancers combined every year from 1988 to 2017. During the time period 2013–2017, the largest number of YLL occurred in lung, bowel and breast cancer, both crude and as age-standardised rates. A steady decline in age-standardised YLL and mortality rates was observed for the entire time period. Calculating YLL using this life table methodology allowed comparisons of YLL by age groups over time. YLL by age for all cancers showed a peak between 60–64 and 75–79. For cervical cancer, on the other hand, we observed two peaks in 1988–1992, one around 30 to 40 and a second one 65–69 years, with the second peak disappearing over time. Furthermore, we observed that AYLL per death increased for some cancers, for example, cervix, and decreased for others, like melanoma skin cancer and leukaemia. Comparing incidence, mortality and YLL in 2013–2017 we observed that some cancers, like prostate, which made up a large proportion of cancer incidence, made up a smaller proportion of mortality and YLL. For other cancers, like pancreas and oesophagus, the proportion of YLL was slightly higher than the proportion of mortality in men.

### Interpretation and significance

The varying trends observed for number of deaths and YLL, i.e., the dip observed at 1998–2002, compared to ASMR and ASYR for the time periods 1988–1992 to 2013–2017 can be explained by an increase in population size due to births, migration and increases in life expectancies resulting in more individuals at older ages [20]. Once the increase in population size, especially at older age groups, is taken into account by age standardisation, a continuous decrease in mortality and YLL becomes apparent.

The differences observed in YLL over time for different cancers overall and for different age groups reflect changes in incidence and mortality rates mostly due to cancer screening and/or new and more effective treatments. For all cancers combined, first a reduction was observed after 1988–1992, however, in 2013–2017 this curve had again returned to the same height. Looking at individual cancers, the biggest changes in YLL by age over the time period 1988 to 2017 were observed for leukaemia, cervical and breast cancer. Even though leukaemia age-standardised incidence rates have increased by around 13% since the early 1990s mortality rates have decreased overall by 12% in females and remained stable in males from the 1970s to 2017–2019. The majority of this mortality reduction was observed at younger ages with a 76% decrease in 0–24 s, 64% in 25–49 s and 55% in 50–59 s [21]. This can be mostly explained by improvements in management, with childhood acute lymphoblastic leukaemia survival rates, for example, now lying around 90% in high-income countries [22]. The impact of this mortality reduction was strongest up to age 25, where YLLs roughly halved. This furthermore resulted in an overall reduction of AYLL from around 17 to around 14. As YLL is most affected by changes in mortality at younger ages and for leukaemia the strongest reduction in mortality was observed in the youngest age group; this explains the strong reduction in YLLs observed here.

For cervical cancer an increase in AYLL was observed over time, however, when looking at YLL by age, in 1988–1992, two peaks were observed, one around 40 years of age and one around 70. The National Health Service (NHS) Cervical Screening Programme was introduced in the UK in 1988 and it was estimated that since then 48% of expected cancers had been prevented with a stronger protective effect at ages from 35 years onwards [23]. Furthermore, Landy et al. showed, using data on 80% of cervical cancer diagnoses in England over 25 years, that the efficacy of screening in preventing cervical cancer mortality (inferred from stage-specific incidence) is much greater at older than at younger ages [24]. In addition, this study estimated that cervical screening prevented 70% of cervical cancer deaths in all ages. This explains both the overall reduction in YLL for all age groups and the stronger reduction of the YLL peak around 70 years. As AYLL estimates the average years one person would have lived had they not died of cancer, the overall increase in AYLL for cervical cancer can also be explained by the higher impact of cervical screening at higher ages. This substantial reduction in cervical cancer deaths at older ages resulted in an increase of the proportion of cancer deaths at younger ages, which in turn led to an increase in AYLL per death from cervical cancer.

For breast cancer, a very slight increase in AYLL with an overall decrease in YLL was observed. The National Health Service Breast Screening Programme (NHSBSP), which invites women for a 3-yearly mammogram from age 50, was also launched in the UK in 1988. Since then the YLL by age peak has reduced in size and has narrowed from a broad peak from age 40–80 in 1988–1992 to a narrower double peak around 50 and 70 years of age. The reduction in size is most likely due to the NHSBSP, which has been found to cause a reduction in mortality and improvements to treatments. The size of mortality reduction is still under debate, however, an extensive review of all breast cancer screening randomised controlled trial estimated it around 20 and a recent case-control study of breast screening in England at 38% [25, 26]. With breast screening starting at 50 the peak of YLL around 50 is probably due to a combination of symptomatic cancers diagnosed before women entered the NHSBSP and aggressive cancers diagnosed in the first round of breast screening. The shift to a peak at an earlier age again explains the slight increase in AYLL, similarly but not as dramatically as for cervical cancer. An increase in AYLL could therefore be caused by an increase in the proportion of deaths to a younger age, however, an increase in life expectance can also contribute. In addition, new treatments, for example endocrine therapies, also started to become used as gold standard in the 1980s and further improved survival [27]. This will have contributed to the overall reduction in YLL between 1988–1992 and 2013–2017.

The NHS bowel screening programme offering guaiac faecal occult blood test started in 2006. A recent population-based study found a reduction in odds of Duke Stage D colorectal cancer, however, it is too early to observe its impact on mortality [28]. As bowel cancer incidence has remained stable since 1993 [29] it is therefore likely that the reduction in ASMR and ASYR from 1988–1992 until 2013–2017 is predominantly due to improvements in treatments/management.

In 2013–2017, in the UK, the cancers with the highest incidence were breast, prostate, lung and bowel cancer (Fig. 4). These cancers combined accounted for over half of all new cancer cases (53%) in 2016–2018 [30]. Comparatively, the cancers with the highest YLL were lung in both sexes, followed by breast and bowel in women, and bowel and prostate in men. Even though prostate cancer had the highest incidence and second-highest mortality in men, it only resulted in the third highest number of YLL, whereas lung and bowel cancer resulted in higher YLL. This is because the number of deaths occurring in bowel and lung cancer are higher in younger age groups compared with the same age groups in prostate cancer [31, 32]. In addition, more prostate cancer deaths occur in older age groups, where life expectancy is lower than compared with younger age groups. For women, breast cancer, which has the highest incidence, resulted both in the second-highest mortality and YLL groups.

This comparison provides helpful insights when determining the most useful measures for resource allocation. Using these three measures researchers, policymakers and funders can obtain a more complete picture of the impact different cancers have on society. These metrics can furthermore be used to better understand the impact different cancer prevention strategies have had over time. Cancer incidence is a particularly good measure to investigate the impact of changes in risk factors prevalence and impact of primary cancer prevention strategies, like smoking legislation. Mortality is a useful measure to observe the long-term impact of early cancer detection interventions and monitoring effectiveness of different cancer screening programmes as well as the success of treatments. However, it can take a long time to observe the impact of new screening programmes. Furthermore, changes in mortality also reflect improvements in treatment and it is not possible to distinguish between the impact of prevention resulting in earlier diagnosis and improvements to treatments. Mortality, unless presented for specific ages, does not take the age at deaths into consideration. In contrast, age at diagnosis included in YLL calculations. It is therefore useful to estimate the impact of different cancers on society or, if looking at AYLL, those dying from cancer. YLL puts a higher weight on cancer deaths in younger individuals, which have a higher impact on society. For example, a young woman dying of cervical cancer might leave behind a young family and therefore representing a higher societal burden. However, as pointed out by one member of the KCL Cancer Prevention public and patient involvement (PPI) group, this has the risk of resulting in ageism, where society values individuals over 70 less. This individual questioned the moral justification of prioritising young with children over older individuals. Other members of the group disagreed with this assessment. When using these measures to make the most relevant decisions for resource allocations, all three measures should be taken into account and ethical decisions regarding the above-mentioned moral issues should be made on a case-by-case basis.

### Relation to other work in the area

Even though the Office of National Statistics in the United Kingdom published age-standardised mortality rates and years of life lost for causes considered avoidable, amenable and preventable for England and Wales for 2018–2020 [33], they only looked at all cancers combined and for ages 1–74 years combined. Using data from the East Anglian Cancer Registry Burnet and colleagues observed lung, breast and bowel cancer to have the highest YLL, followed by stomach, prostate and ovary in 1990–1994 [11]. Similarly, we also found the first five cancers to have the highest YLL in the time period 1988–1992, however, we estimated the YLL for ovarian cancer to be less than for the pancreas and oesophagus. These differences might be due to comparing a single area in England with the whole of the UK. This decrease in YLL from stomach cancer is most likely due to a large reduction in incidence due to a combination of reduction in smoking and *H. pylori* infections [34]. Two recent studies analysing PYLL due to cancer in the United States in 2017 and YLL in Norway in 2012 also found that the largest numbers of PYLL and YLL occurred from lung and bronchus, colorectum and breast followed by pancreatic cancer [17, 35], same as we presented here. However, some differences in the highest AYLL were observed. Of the 17 cancers analysed in the UK for 2013–2017 the highest AYLL were observed for the cervix, ovary, melanoma skin cancer, leukaemia and breast, whereas in the US, amongst the same cancers we analysed cancers of the cervix and leukaemia were found to have highest AYLL. As the US study estimated PYLL before age 75, it is not possible to directly compare PYLL and YLL values. The Norway study on the other hand observed very similar AYLL, with cervix being the highest about 20 followed by melanoma, ovarian and breast cancer. Only leukaemia was estimated lower as the cancer with the 10th highest AYLL. This might be due to changes in treatments between Norway and the UK.

The global burden of disease (GBD) study estimated YLL for 29 cancers for every country worldwide from 2010 to 2019, including for the UK [10]. Comparing their mortality and YLL data, which is freely available online [36], for the time period 2013 to 2017 for several cancers, GBD consistently estimate much higher number of deaths and YLL than we do. For example, for breast cancer, the GBD number of deaths and YLL for 2017 were 14,640 and 293,627, respectively, compared to 11,435 deaths and 196,885 YLL for 2013–2017 in our study. Similarly, the GBD number of colorectal cancer deaths and YLL for 2017 were 22,815 and 385,195 compared to 16,133 and 213,535 for 2013–2017 in our study. The GBD Cancer Collaboration used a series of models to estimate mortality. By contrast, the mortality data we use was obtained directly from the four cancer registries in the UK and reflect actual numbers of death certificates with cause of death recorded as cancer. It could be argued that excess deaths in cancer patients better reflects the true mortality from cancer than using cause of death [37], but the difference in resulting mortality in the UK are likely to be small as the ONS uses strict selection rules to code cause of death as precisely as possible. Thus, it seems that the GBD models substantially overestimate mortality and therefore cannot be relied upon for YLL. The GBD Cancer Collaboration also used a different methodology to calculate YLL. They calculate YLL by subtracting the age at death from the longest possible life expectancy ignoring cancer deaths after that age [8]. By contrast, we estimate the residual life expectancy at the time of each cancer death following the definition of the World Health Organisation [9]. In addition, they used standard GBD life expectancy tables, whereas we used UK-specific life tables calculated by the ONS in.

### Strength and limitations

The present study uses population-based data on all registered cancer deaths for all four countries in the UK to estimate YLL to cancer covering a 30-year time period from 1988 to 2017. We not only analysed 17 different cancers in detail but also estimated YLL and mortality for these cancer sites by sex and for different age groups. As this study has included data from 1988 to 2017, it is possible to observe trends during a time period that saw the introduction of all NHS cancer screening programmes, changes to cancer treatments and changes to some cancer risk and protective factors. We use a simple methodology that could be easily replicated by cancer registries or charities that have direct access to mortality data.

This study has two potential limitations though. Firstly, as life expectancy calculations include deaths due to cancer, it is not possible to estimate the true YLL due to cancer using the methodology used here. However, the aim of this study was to provide an extensive analysis of YLL for the most common cancers over a time span of 30 years using the most up-to-date data for the whole of the UK for researchers, policymakers and other stakeholders to use. As the same methodology was used throughout this study and other published studies, it is possible to carry out comparisons. Furthermore, we are planning to address the issue of true YLL using more complex statistical methodology in a future study. Secondly, using data from the ONS, Northern Ireland Cancer Registry and ISD Scotland, data quality could impact study findings. For example, in a small number of cases, cause of death might not have been coded correctly resulting in some cancers being missed. When identifying the underlying cause of death, however, the ONS uses strict selection rules which are applied uniformly, so that cause of death with be coded as precisely as possible and data will be comparable between different times and places [38].

### Main conclusion and future direction

YLL is a useful measure that can increase the prominence of certain cancers, such as cervical cancer, that predominantly affect younger age groups. AYLL, which looks at YLL per death for specific cancer, is particularly useful for identifying cancers with the worst impact on patientsʼ lives. Comparing changes in YLL with changes in mortality rates can highlight when the age distribution of cancer is changing over time. Using YLL in addition to mortality will provide a more accurate basis for health policy discussions, public health decisions, and research funding allocation. AYLL could be a particularly useful measure for easy comparisons of different cancers by policymakers and the general public.

### Supplementary information

YLL Supplementary Materials

Supplementary Figure 1

Supplementary Table 1

Supplementary Table 2

## Data Availability

For publically available data, we have provided references with URLs for data access in the methods section. With regard to cancer data obtained from national cancer registries, we are not able to share data with third parties. We are happy to share our detailed data applications with other researchers upon request, so that the same data can be obtained from registries.

## References

[1] GBD 2016 Causes of Death Collaborators. (2017). Global, regional, and national age-sex specific mortality for 264 causes of death, 1980-2016: a systematic analysis for the Global Burden of Disease Study 2016. Lancet.

[2] Cancer Research UK. Cancer mortality statistics, https://www.cancerresearchuk.org/health-professional/cancer-statistics/mortality#heading-Zero. Accessed 21 November 2021.

[3] Estève J, Benhamou E, Raymond L. Statistical methods in cancer research. Volume IV. Descriptive epidemiology. IARC Sci Publ. 1994;128:302. 7698823

[4] Miller KD, Fidler-Benaoudia M, Keegan TH, Hipp HS, Jemal A, Siegel RL (2020). Cancer statistics for adolescents and young adults, 2020. CA Cancer J Clin.

[5] Cancer Research UK. Cancer Incidence by Age, https://www.cancerresearchuk.org/health-professional/cancer-statistics/incidence/age#heading-Zero. Accessed 25 August 2022.

[6] Gardner JW, Sanborn JS (1990). Years of potential life lost (YPLL)-what does it measure?. Epidemiology.

[7] Møller H, Fairley L, Coupland V, Okello C, Green M, Forman D (2007). The future burden of cancer in England: incidence and numbers of new patients in 2020. Br J Cancer.

[8] Chudasama YV, Khunti K, Gillies CL, Dhalwani NN, Davies MJ, Yates T (2022). Estimates of years of life lost depended on the method used: tutorial and comparative investigation. J Clin Epidemiol.

[9] The Global Health Observation. *Y*ears of life lost (YLL) (per 100 000 population), https://www.who.int/data/gho/indicator-metadata-registry/imr-details/4427. Accessed 21 November 2021.

[10] Global Burden of Disease Cancer Collaboration. (2022). Cancer incidence, mortality, years of life lost, years lived with disability, and disability-adjusted life years for 29 cancer groups from 2010 to 2019: a systematic analysis for the global burden of disease study 2019. JAMA Oncol.

[11] Burnet NG, Jefferies SJ, Benson RJ, Hunt DP, Treasure FP (2005). Years of life lost (YLL) from cancer is an important measure of population burden—and should be considered when allocating research funds. Br J Cancer.

[12] Office for National Statistics. National life tables, United Kingdom, 1980–82 to 2014–16. Dataset. Released 27 September 2017. https://www.ons.gov.uk/peoplepopulationandcommunity/birthsdeathsandmarriages/lifeexpectancies/datasets/nationallifetablesunitedkingdomreferencetables/current.

[13] Office for National Statistics. Guide to calculating national life tables. 2019. https://www.ons.gov.uk/peoplepopulationandcommunity/healthandsocialcare/healthandlifeexpectancies/methodologies/guidetocalculatingnationallifetables#:~:text=A%20life%20table%20is%20a%20hypothetical%20calculation%2C%20the%20national%20life,before%20the%20three%2Dyear%20period. Accessed 21 November 2021.

[14] Martinez R, Soliz P, Caixeta R, Ordunez P (2019). Reflection on modern methods: years of life lost due to premature mortality—a versatile and comprehensive measure for monitoring non-communicable disease mortality. Int J Epidemiol.

[15] Parkin DM, Pisani P, Ferlay J (1999). Global cancer statistics. CA Cancer J Clin.

[16] Eurostat European Comission. Revision of the European Standard Population—Report of Eurostat’s task force—2013 edition. Luxembourg: Publications Office of the European Union; 2013.

[17] Brustugun OT, Møller B, Helland A (2014). Years of life lost as a measure of cancer burden on a national level. Br J Cancer.

[18] Zhang D (2017). A coefficient of determination for generalized linear models. Am Stat.

[19] R Core Team. R: a language and environment for statistical computing. R Foundation for Statistical Computing, Vienna, Austria. https://www.R-project.org/; 2021.

[20] Crofts S, Stripe N. Our population—where are we? How did we get here? Where are we going?, Office of National Statistics; 2020. https://www.ons.gov.uk/peoplepopulationandcommunity/populationandmigration/populationestimates/articles/ourpopulationwherearewehowdidwegetherewherearewegoing/2020-03-27; Accessed 21 November 2021.

[21] Cancer Research UK. Leukaemia (all subtypes combined) mortality statistics. https://www.cancerresearchuk.org/health-professional/cancer-statistics/statistics-by-cancer-type/leukaemia/mortality#ref-2. Accessed 25 August 2022.

[22] Pui CH, Evans WE (2013). A 50-year journey to cure childhood acute lymphoblastic leukemia. Semin Hematol.

[23] Pesola F, Sasieni P (2019). Impact of screening on cervical cancer incidence in England: a time trend analysis. BMJ Open.

[24] Landy R, Pesola F, Castañón A, Sasieni P (2016). Impact of cervical screening on cervical cancer mortality: estimation using stage-specific results from a nested case-control study. Br J Cancer.

[25] Marmot MG, Altman DG, Cameron DA, Dewar JA, Thompson SG, Wilcox M (2013). The benefits and harms of breast cancer screening: an independent review: a report jointly commissioned by Cancer Research UK and the Department of Health (England) October 2012. Br J Cancer.

[26] Maroni R, Massat NJ, Parmar D, Dibden A, Cuzick J, Sasieni PD (2021). A case-control study to evaluate the impact of the breast screening programme on mortality in England. Br J Cancer.

[27] Sainsbury R (2013). The development of endocrine therapy for women with breast cancer. Cancer Treat Rev.

[28] Castanon A, Parmar D, Massat NJ, Sasieni P, Duffy SW (2022). Benefit of biennial fecal occult blood screening on colorectal cancer in England: a population-based case-control study. J Natl Cancer Inst.

[29] Cancer Research UK. Bowel cancer incidence statistics. https://www.cancerresearchuk.org/health-professional/cancer-statistics/statistics-by-cancer-type/bowel-cancer/incidence#heading-Two. Accessed 3 July 2023.

[30] Cancer Research UK. Cancer Incidence Statistics. https://www.cancerresearchuk.org/health-professional/cancer-statistics/incidence#heading-Two. Accessed 25 August 2022.

[31] Cancer Research UK. Lung cancer mortality statistics. https://www.cancerresearchuk.org/health-professional/cancer-statistics/statistics-by-cancer-type/lung-cancer/mortality#heading-One. Accessed 20 August 2022.

[32] Cancer Research UK. Bowel cancer mortality statistics. https://www.cancerresearchuk.org/health-professional/cancer-statistics/statistics-by-cancer-type/bowel-cancer/mortality. Accessed 1 November 2022.

[33] Office of National Statistics. Age-standardised mortality rates and years of life lost for causes considered avoidable, amenable and preventable for England and Wales, and English regions. 2017. Dateset. Released 6 April 2017. https://service-manual.ons.gov.uk/content/formatting-and-punctuation/citations-references-and-sources

[34] Lin Y, Zheng Y, Wang H-L, Wu J (2021). Global patterns and trends in gastric cancer incidence rates (1988–2012) and predictions to 2030. Gastroenterology.

[35] Song M, Hildesheim A, Shiels MS (2020). Premature years of life lost due to cancer in the United States in 2017. Cancer Epidemiol Biomark Prev.

[36] Global Burden of Disease Cancer Collaboration. GDB Results. https://vizhub.healthdata.org/gbd-results/. Accessed 1 July 2019.

[37] Bright CJ, Brentnall AR, Wooldrage K, Myles J, Sasieni P, Duffy SW (2020). Errors in determination of net survival: cause-specific and relative survival settings. Br J Cancer.

[38] Woods R, Cooke A. User guide to mortality statistics. Office of National Statistics. https://www.ons.gov.uk/peoplepopulationandcommunity/birthsdeathsandmarriages/deaths/methodologies/userguidetomortalitystatisticsjuly2017. Accessed 30 August 2022.

