# Supplementary material for: Years of life lost due to cancer in the United Kingdom from 1988 to 2017
Source: Br J Cancer. 2023 Sep 19;129(10):1558–68. doi: 10.1038/s41416-023-02422-8 (PMC10645733; doi:10.1038/s41416-023-02422-8)

**Supplementary Materials**

**Figures**

**Supplementary Figure 1:** YLL by age group from 1988-1992 to 2013-2017. Graphs for all 17 cancers.

See separate PDF file.

**Supplementary Figure 2**: Annual average percent change between 1988-1992 and 2013-2017 for 17 cancer sites and the all cancers combined.


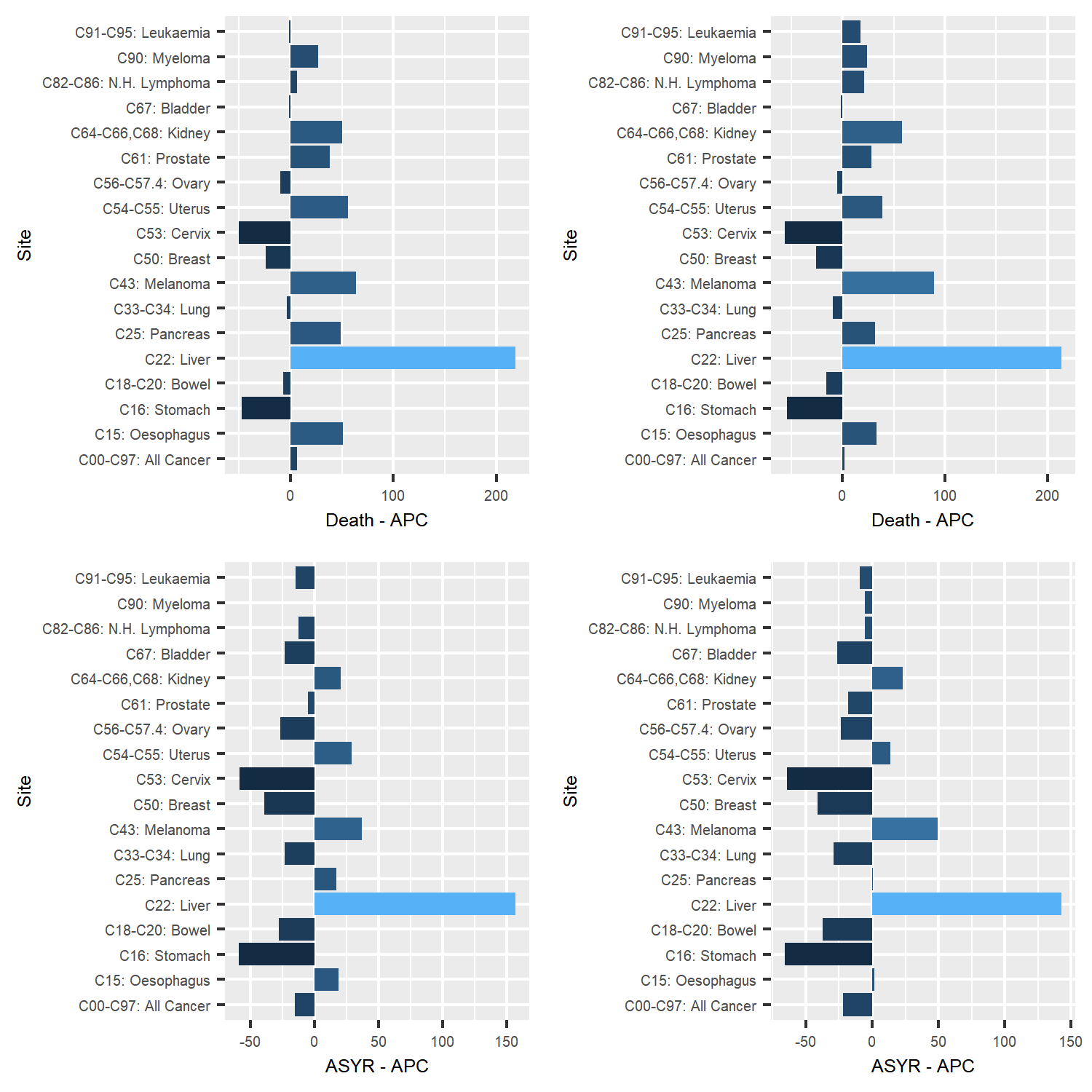


**Tables**

Supplementary Tables 1 and 2, see separate Excel files.

**Supplementary Table 3:** Annual change in age standardised years of life lost rate (ASYR) and age standardised mortality rate (ASMR) from 1988 to 2017.

| **Cancer Site** | **Annual change* in ASYR (95% CI)** | **Annual change* ASMR (95% CI)** |
| --- | --- | --- |
| **All Cancers C00-C97** | -0.0306 (-0.0360, -0.0253) | -0.0493 (-0.0524, -0.0462) |
| **C33-C34: Lung** | -0.0485 (-0.0327, -0.0643) | -0.0666 (-0.0792, -0.0541) |
| **C50: Breast** | -0.0985 (-0.0933, -0.1038) | -0.1059 (-0.1109, -0.1008) |
| **C61: Prostate** | -0.0104 (0.0004, -0.0213) | -0.0427 (-0.0556, -0.0298) |
| **C18-C20: Bowel** | -0.0632 (-0.0488, -0.0777) | -0.0934 (-0.106, -0.0808) |
| **C56-C57.4: Ovary** | -0.0618 (-0.0452, -0.0785) | -0.0529 (-0.0736, -0.0322) |
| **C25: Pancreas** | 0.0406 (0.0606, 0.0205) | 0.0070 (-0.0114, 0.0255) |
| **C15: Oesophagus** | 0.0341 (0.0564, 0.0118) | 0.0008 (-0.0222, 0.0238) |
| **C22: Liver** | 0.1935 (0.2150, 0.1720) | 0.1775 (0.1558, 0.1992) |
| **C91-C95: Leukaemia** | -0.0272 (-0.016, -0.0384) | -0.0117 (-0.0238, 0.0004) |
| **C82-C86: Non-Hodgkin Lymphoma** | -0.0347 (-0.0066, -0.0628) | -0.0173 (-0.0388, 0.0043) |
| **C64-C66,C68: Kidney** | 0.0418 (0.0531, 0.0304) | 0.0459 (0.0363, 0.0555) |
| **C54-C55: Uterus** | 0.0649 (0.1069, 0.0230) | 0.0356 (-0.005, 0.0762) |
| **C16: Stomach** | -0.1817 (-0.1681, -0.1953) | -0.2168 (-0.225, -0.2086) |
| **C67: Bladder** | -0.0531 (-0.0395, -0.0668) | -0.0648 (-0.0772, -0.0524) |
| **C53: Cervix** | -0.1849 (-0.1366, -0.2332) | -0.2135 (-0.2473, -0.1798) |
| **C43: Melanoma Skin Cancer** | 0.0623 (0.0832, 0.0414) | 0.0759 (0.0581, 0.0937) |
| **C90: Myeloma** | 0.0001 (0.0048, -0.0046) | -0.0124 (-0.019, -0.0058) |

CI, Confidence Interval; ASYR, age standardised years of life lost rate; ASMR, age standardised mortality rate.

*estimated as log(relative rate)

**Supplementary Table 4:** Average YLL of cancer estimated for six time-periods by sex. The average YLL was computed by dividing the total YLL by the total mortality, e.g., the average YLL from the lung cancer (C33-C34) at year 2013-2017 was computed as flowing 500608.86/35485 = 14.10762.


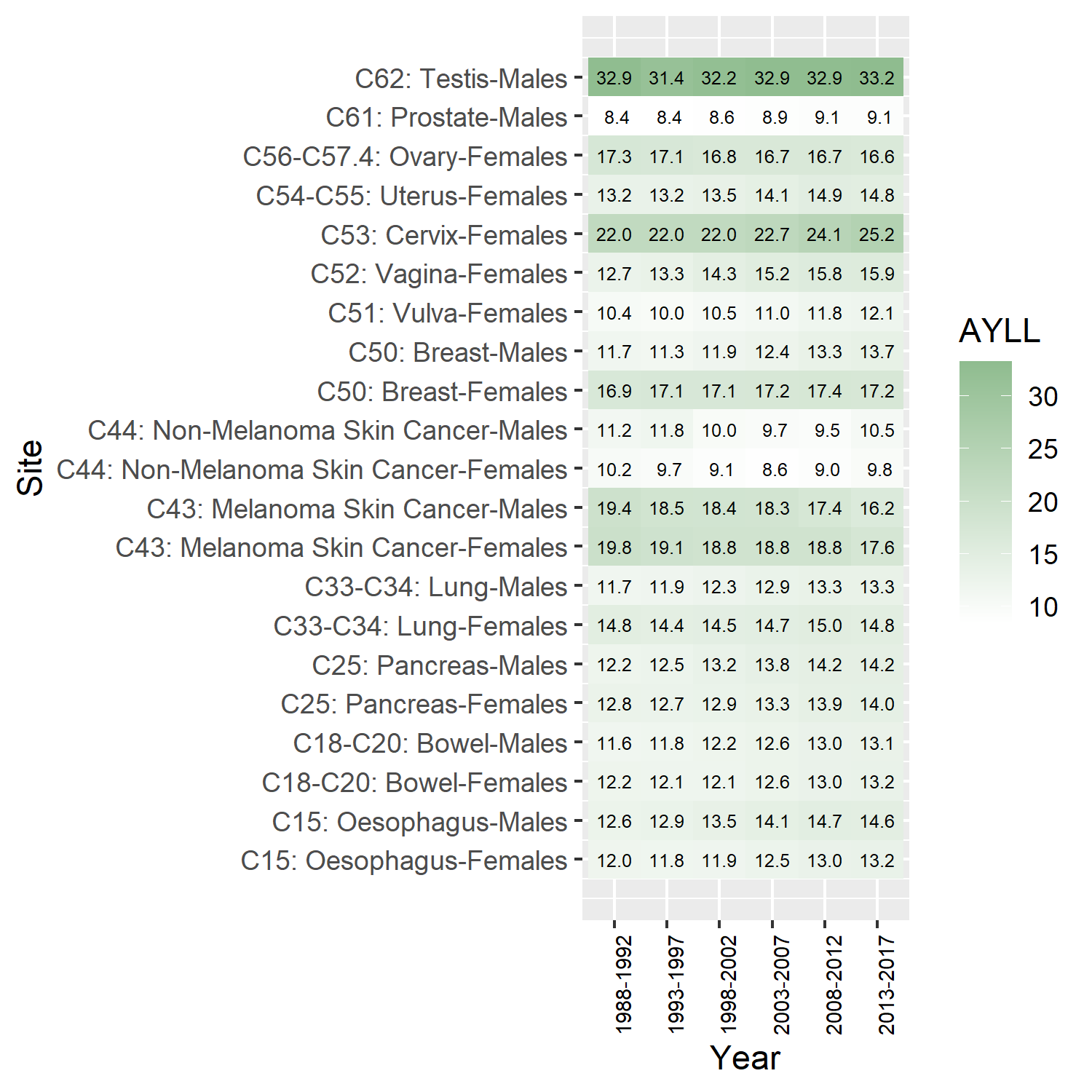

Supplement: Supplementary file 1 — YLL Supplementary Materials [file 41416_2023_2422_MOESM1_ESM.docx]
