# Supplementary material for: Years of life lost due to cancer in the United Kingdom from 1988 to 2017
Source: Br J Cancer. 2023 Sep 19;129(10):1558–68. doi: 10.1038/s41416-023-02422-8 (PMC10645733; doi:10.1038/s41416-023-02422-8)
Supplement: Supplementary file 2 — Supplementary Figure 1 [file 41416_2023_2422_MOESM2_ESM.pdf]

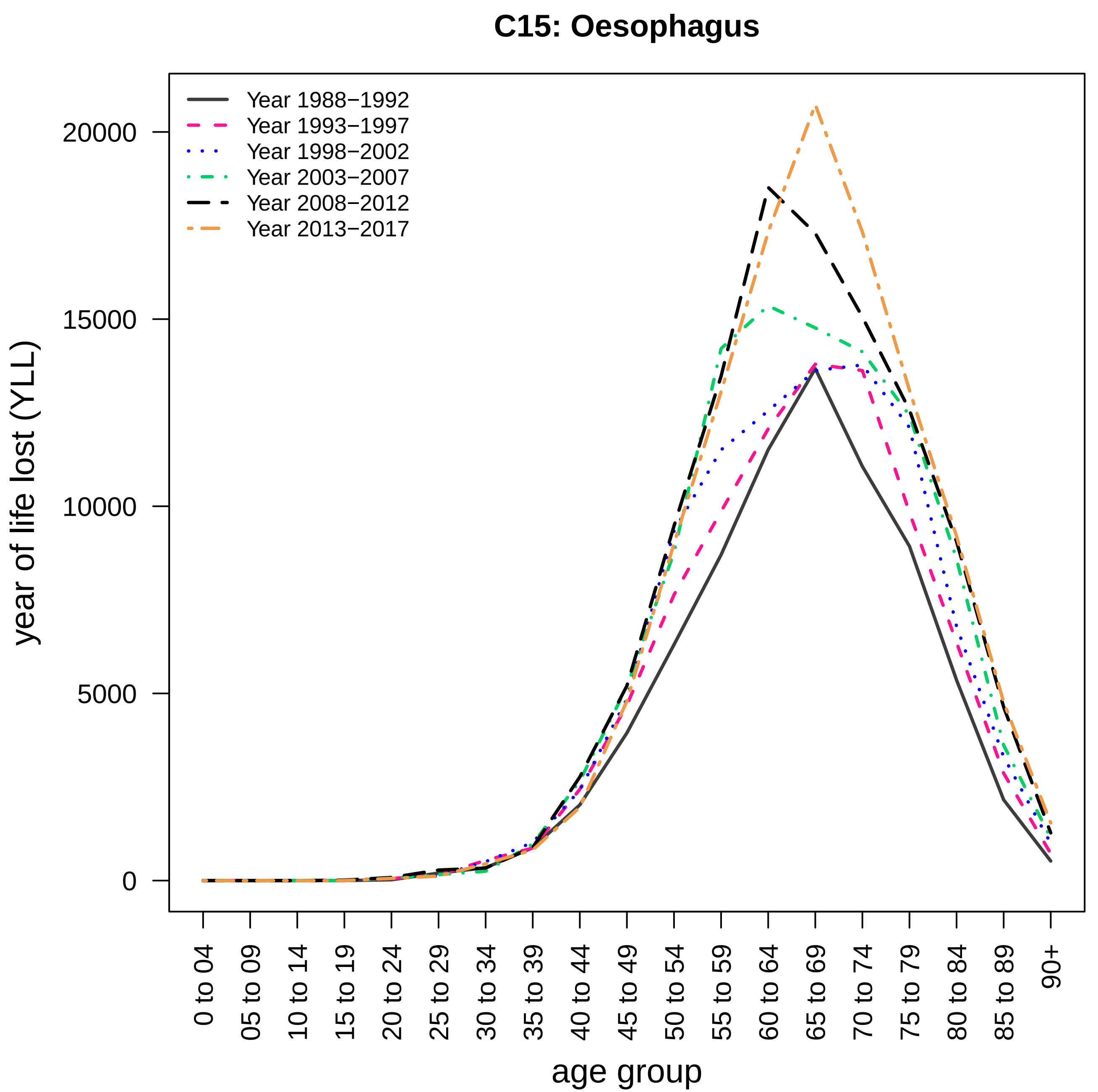
C15: Oesophagus
Year 1988−1992
Year 1993−1997
Year 1998−2002
Year 2003−2007
Year 2008−2012
Year 2013−2017
year of life lost (YLL)
0
5000
10000
15000
20000
0 to 04
05 to 09
10 to 14
15 to 19
20 to 24
25 to 29
30 to 34
35 to 39
40 to 44
45 to 49
50 to 54
55 to 59
60 to 64
65 to 69
70 to 74
75 to 79
80 to 84
85 to 89
90+
age group

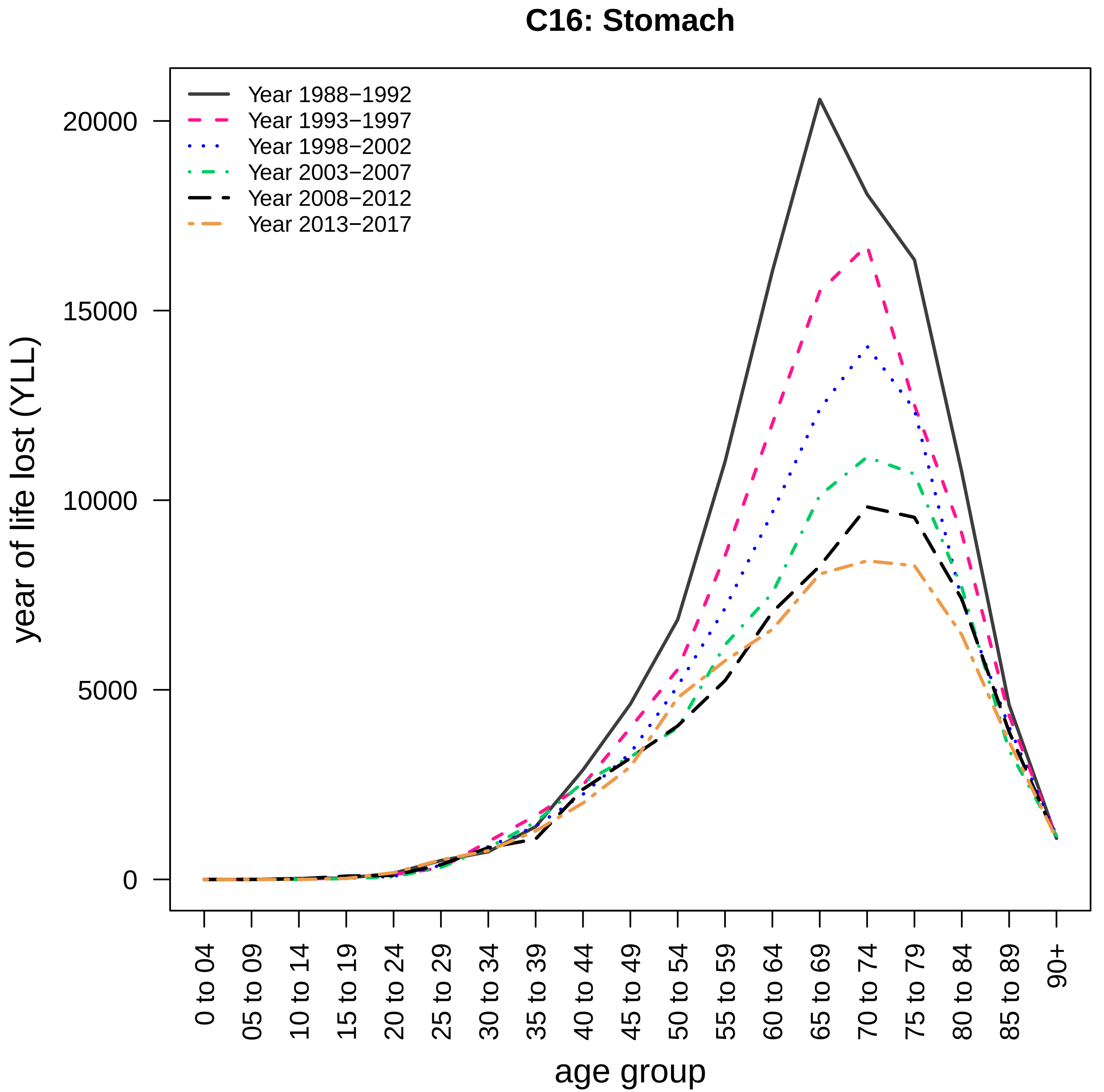
C16: Stomach
Year 1988−1992
Year 1993−1997
Year 1998−2002
Year 2003−2007
Year 2008−2012
Year 2013−2017
year of life lost (YLL)
0
5000
10000
15000
20000
0 to 04
05 to 09
10 to 14
15 to 19
20 to 24
25 to 29
30 to 34
35 to 39
40 to 44
45 to 49
50 to 54
55 to 59
60 to 64
65 to 69
70 to 74
75 to 79
80 to 84
85 to 89
90+
age group

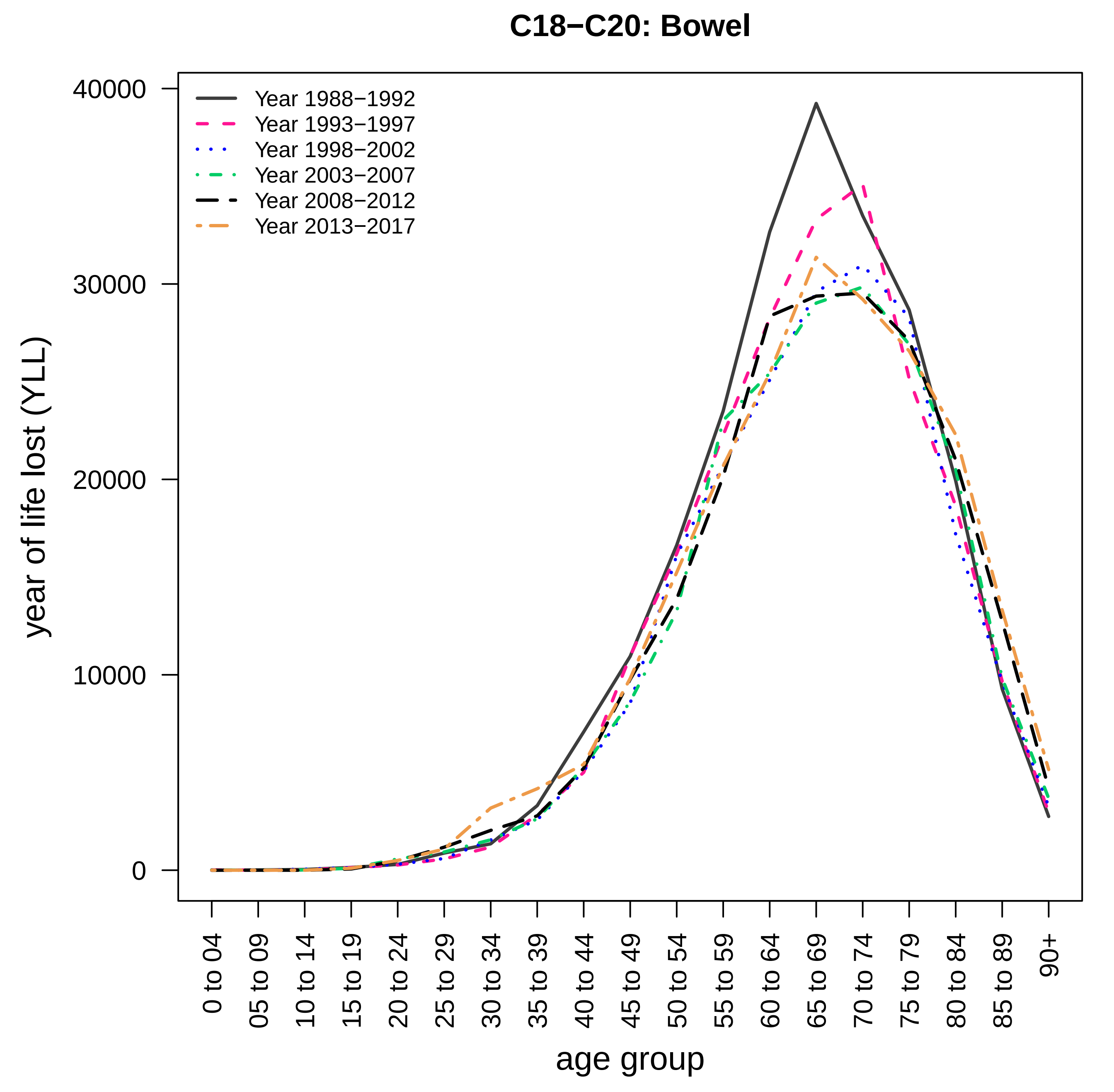

C18−C20: Bowel
Year 1988−1992
Year 1993−1997
Year 1998−2002
Year 2003−2007
Year 2008−2012
Year 2013−2017
year of life lost (YLL)
40000
30000
20000
10000
0
0 to 04
05 to 09
10 to 14
15 to 19
20 to 24
25 to 29
30 to 34
35 to 39
40 to 44
45 to 49
50 to 54
55 to 59
60 to 64
65 to 69
70 to 74
75 to 79
80 to 84
85 to 89
90+
age group

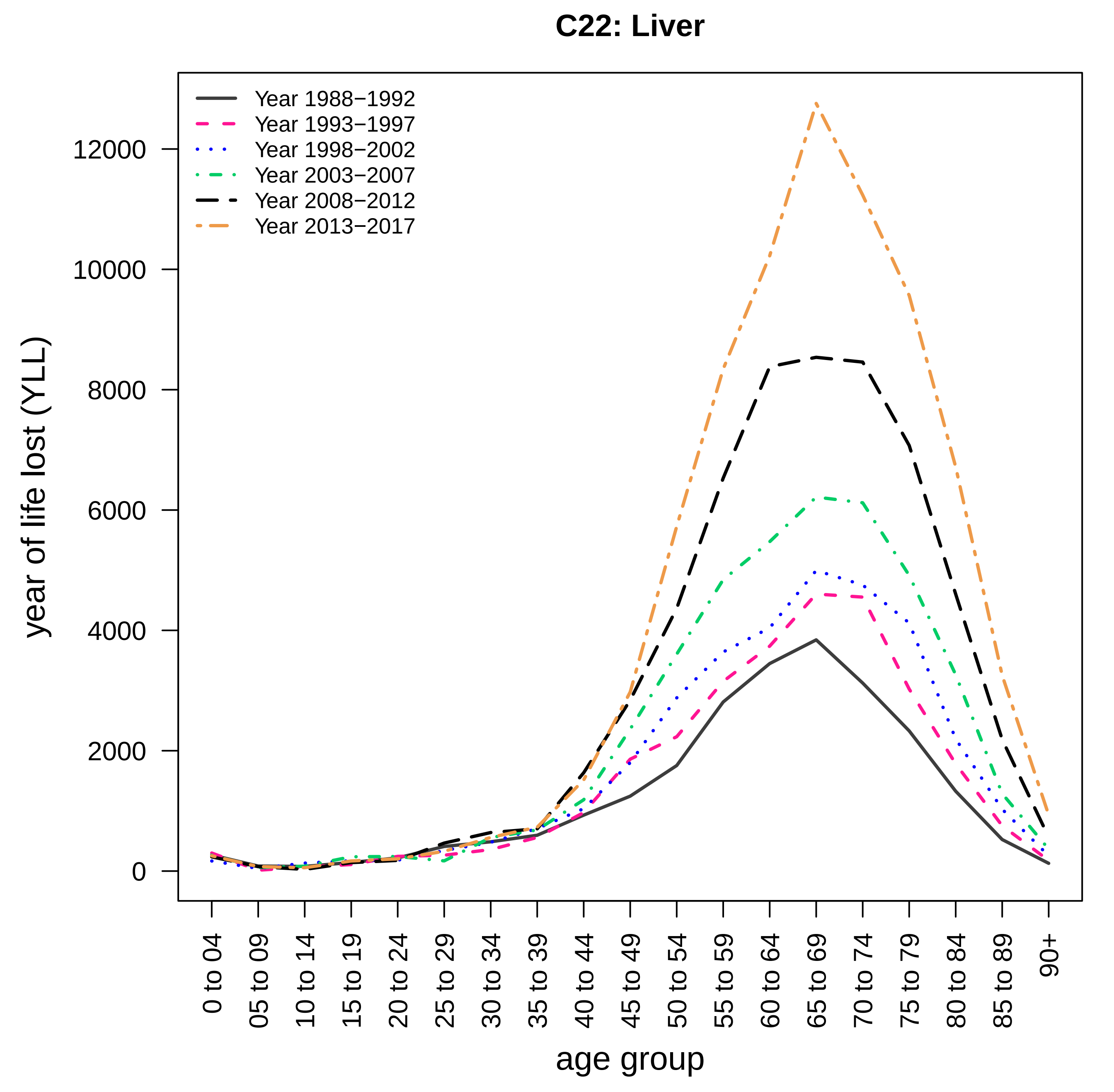

C22: Liver
Year 1988−1992
Year 1993−1997
Year 1998−2002
Year 2003−2007
Year 2008−2012
Year 2013−2017
year of life lost (YLL)
0
2000
4000
6000
8000
10000
12000
0 to 04
05 to 09
10 to 14
15 to 19
20 to 24
25 to 29
30 to 34
35 to 39
40 to 44
45 to 49
50 to 54
55 to 59
60 to 64
65 to 69
70 to 74
75 to 79
80 to 84
85 to 89
90+
age group

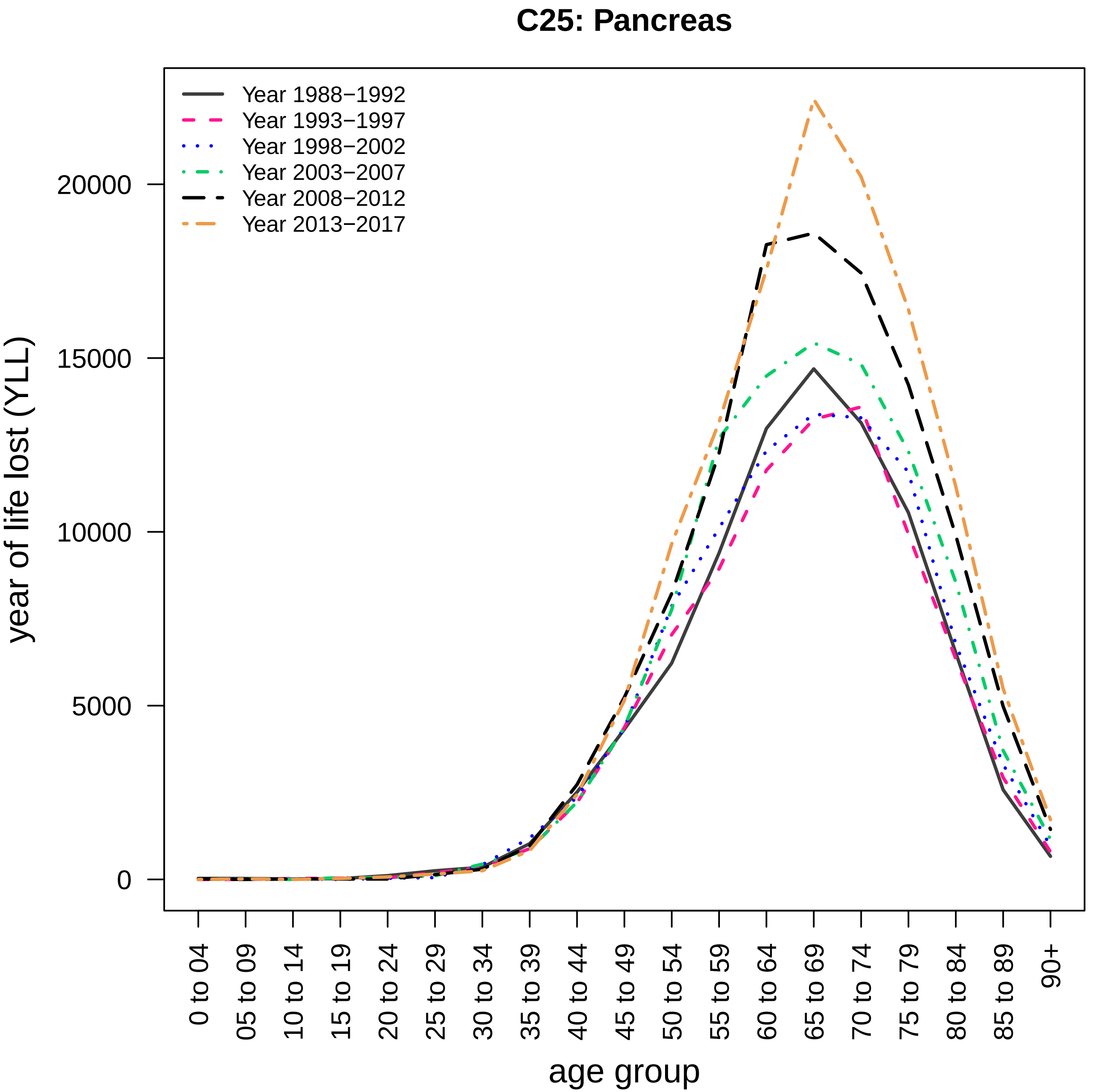
C25: Pancreas
Year 1988−1992
Year 1993−1997
Year 1998−2002
Year 2003−2007
Year 2008−2012
Year 2013−2017
year of life lost (YLL)
0
5000
10000
15000
20000
0 to 04
05 to 09
10 to 14
15 to 19
20 to 24
25 to 29
30 to 34
35 to 39
40 to 44
45 to 49
50 to 54
55 to 59
60 to 64
65 to 69
70 to 74
75 to 79
80 to 84
85 to 89
90+
age group

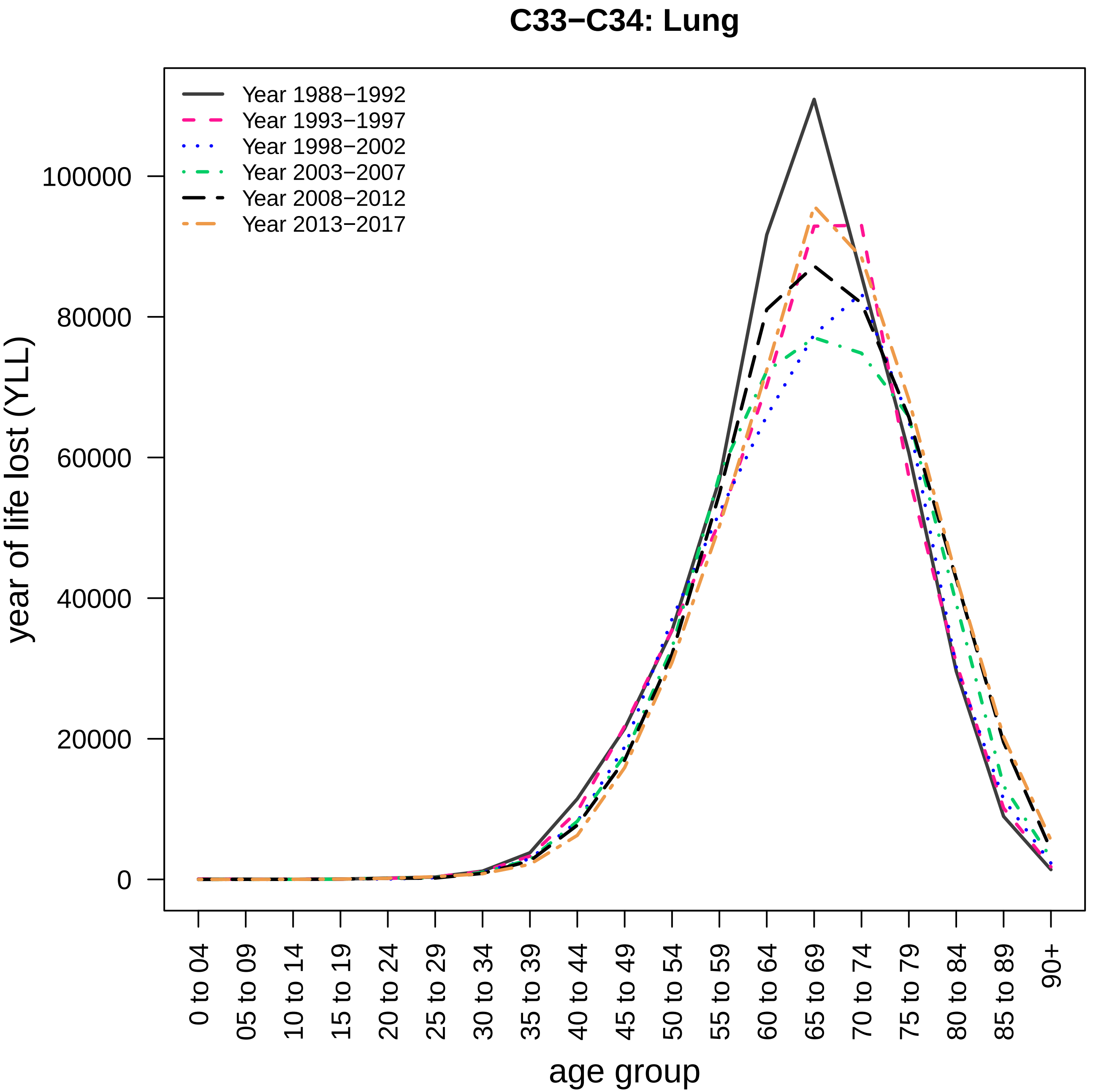

C33−C34: Lung
Year 1988−1992
Year 1993−1997
Year 1998−2002
Year 2003−2007
Year 2008−2012
Year 2013−2017
year of life lost (YLL)
0
20000
40000
60000
80000
100000
0 to 04
05 to 09
10 to 14
15 to 19
20 to 24
25 to 29
30 to 34
35 to 39
40 to 44
45 to 49
50 to 54
55 to 59
60 to 64
65 to 69
70 to 74
75 to 79
80 to 84
85 to 89
90+
age group

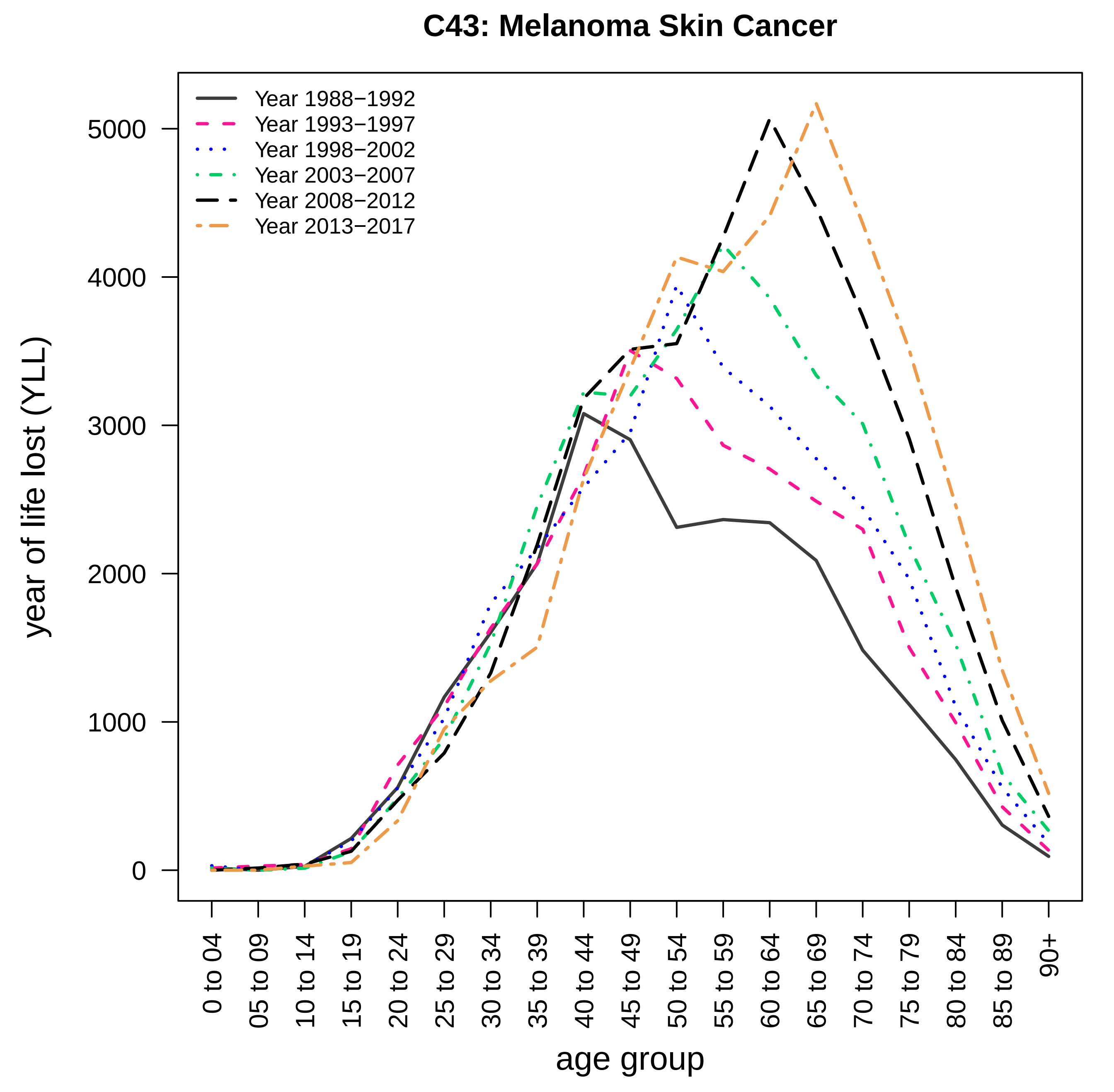
C43: Melanoma Skin Cancer
Year 1988−1992
Year 1993−1997
Year 1998−2002
Year 2003−2007
Year 2008−2012
Year 2013−2017
year of life lost (YLL)
0
1000
2000
3000
4000
5000
0 to 04
05 to 09
10 to 14
15 to 19
20 to 24
25 to 29
30 to 34
35 to 39
40 to 44
45 to 49
50 to 54
55 to 59
60 to 64
65 to 69
70 to 74
75 to 79
80 to 84
85 to 89
90+
age group

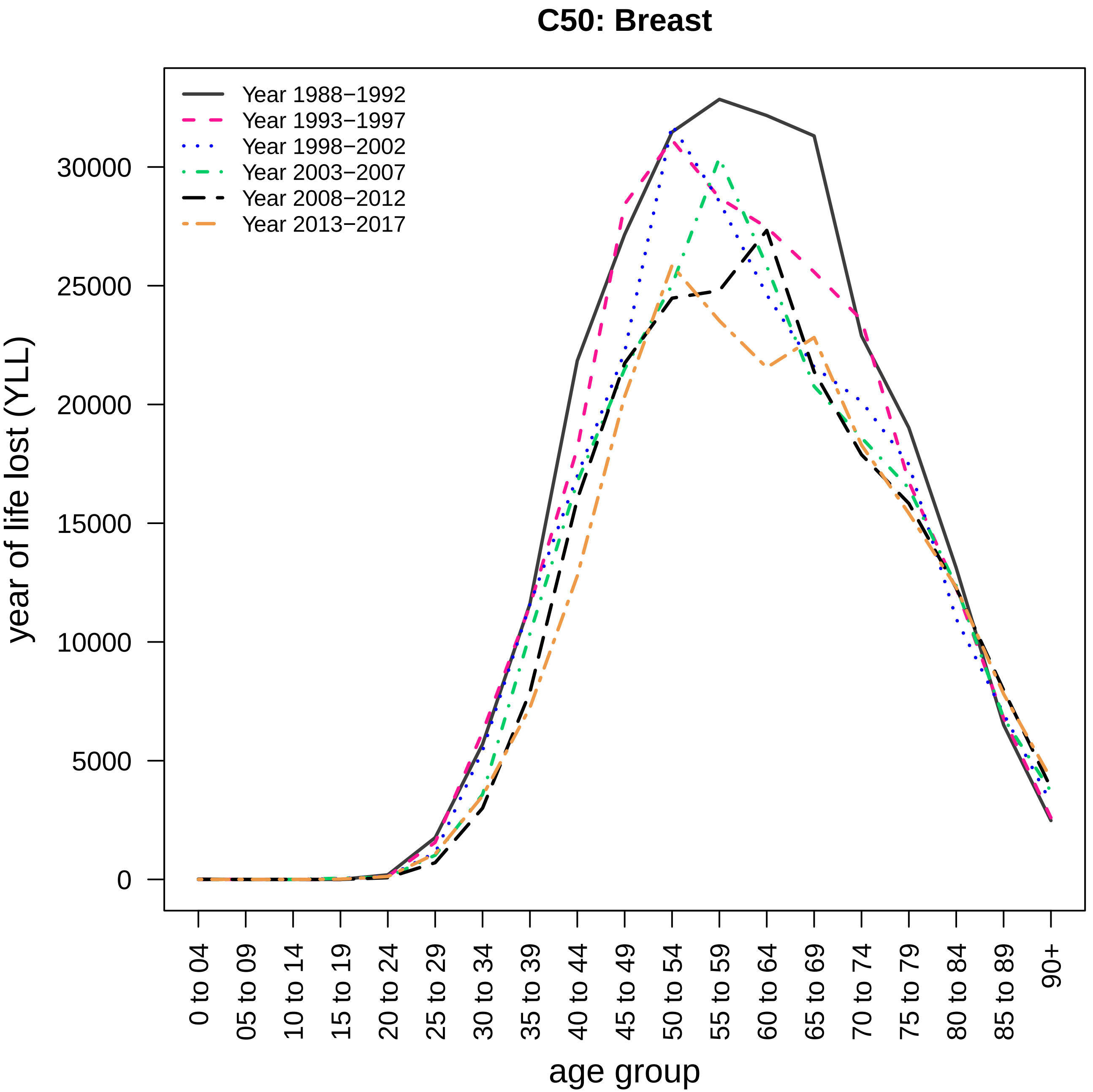
C50: Breast
Year 1988−1992
Year 1993−1997
Year 1998−2002
Year 2003−2007
Year 2008−2012
Year 2013−2017
year of life lost (YLL)
0
5000
10000
15000
20000
25000
30000
0 to 04
05 to 09
10 to 14
15 to 19
20 to 24
25 to 29
30 to 34
35 to 39
40 to 44
45 to 49
50 to 54
55 to 59
60 to 64
65 to 69
70 to 74
75 to 79
80 to 84
85 to 89
90+
age group

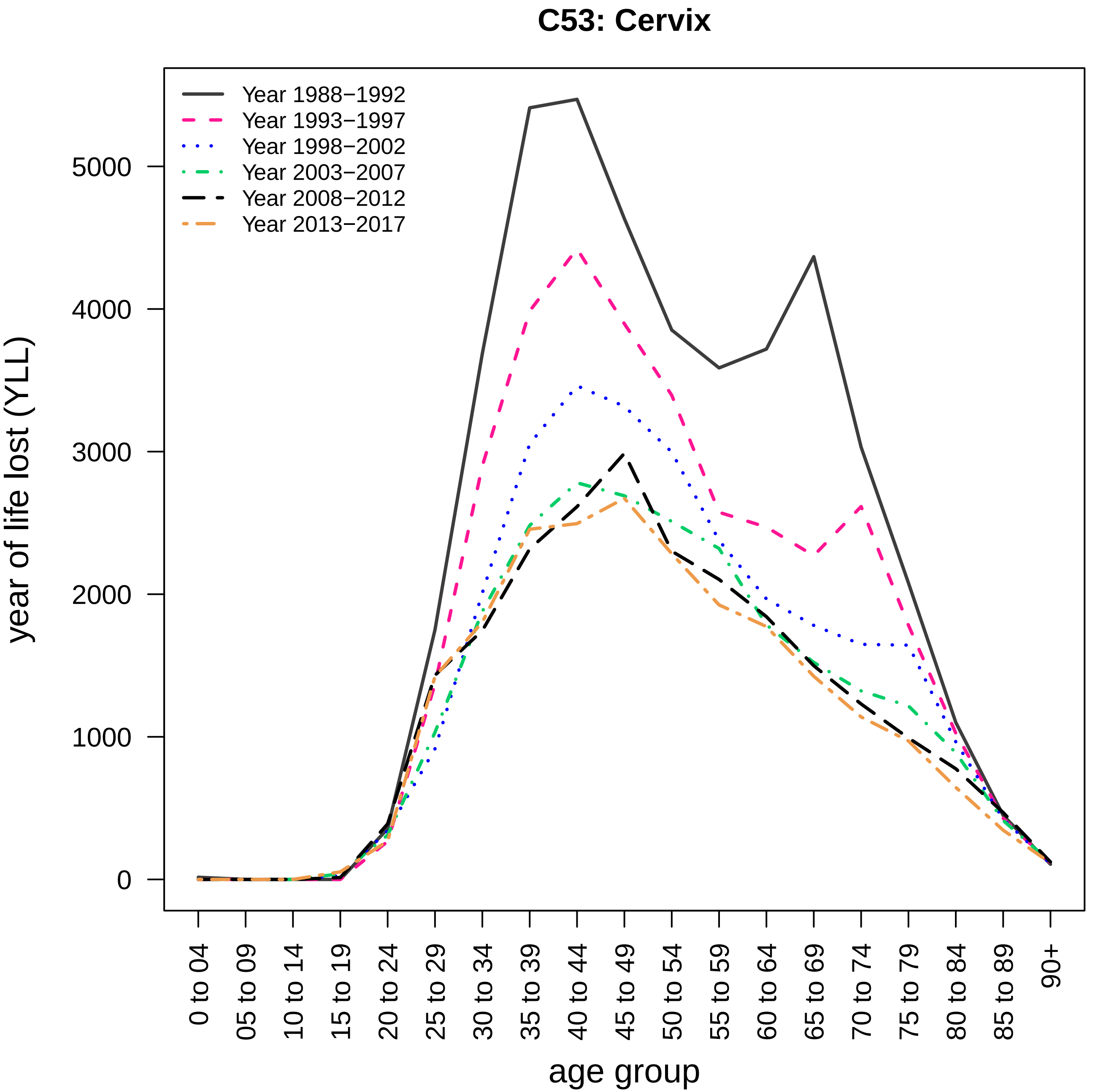
C53: Cervix
Year 1988−1992
Year 1993−1997
Year 1998−2002
Year 2003−2007
Year 2008−2012
Year 2013−2017
year of life lost (YLL)
0
1000
2000
3000
4000
5000
0 to 04
05 to 09
10 to 14
15 to 19
20 to 24
25 to 29
30 to 34
35 to 39
40 to 44
45 to 49
50 to 54
55 to 59
60 to 64
65 to 69
70 to 74
75 to 79
80 to 84
85 to 89
90+
age group

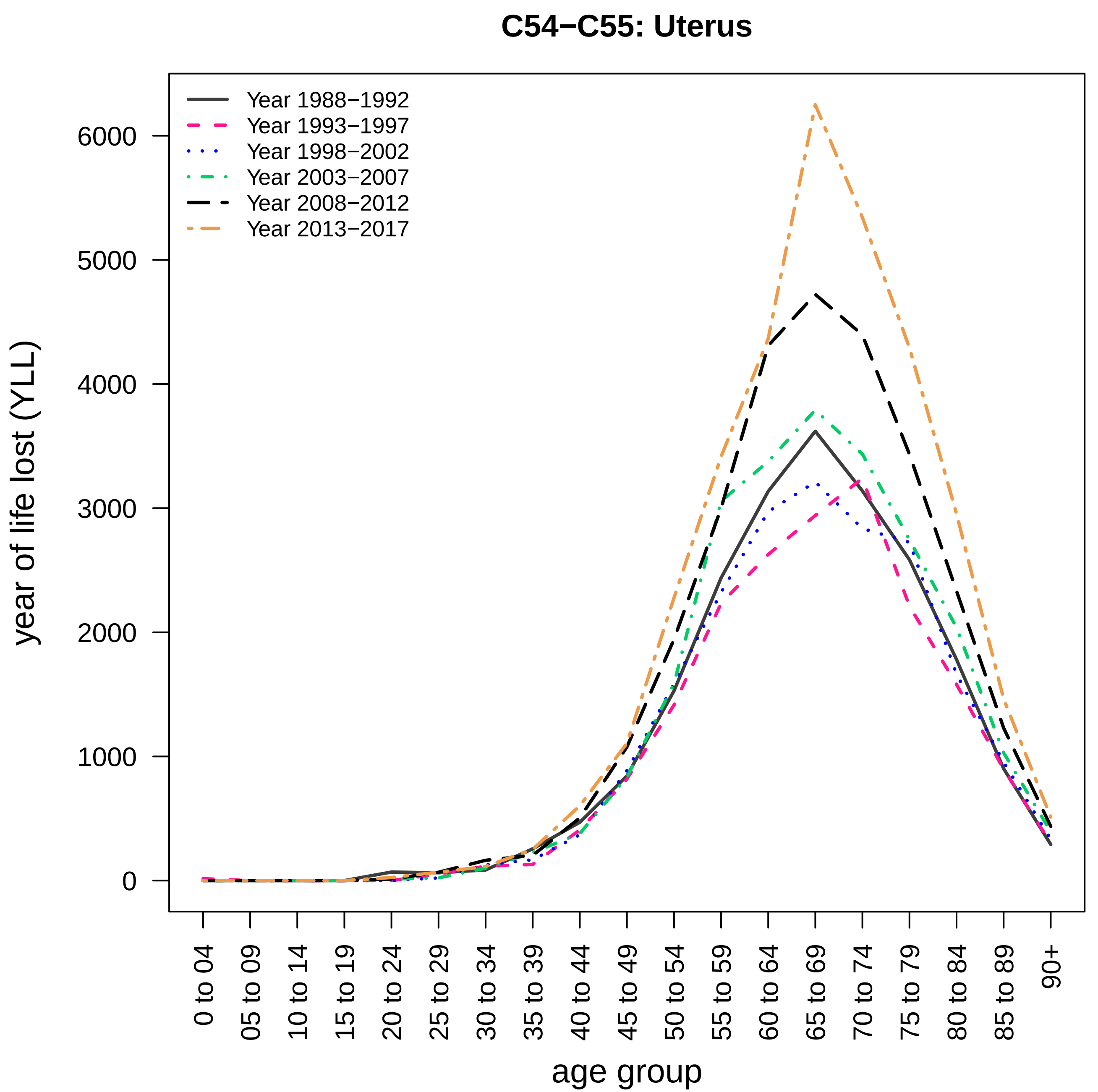

C54−C55: Uterus
Year 1988−1992
Year 1993−1997
Year 1998−2002
Year 2003−2007
Year 2008−2012
Year 2013−2017
year of life lost (YLL)
0
1000
2000
3000
4000
5000
6000
0 to 04
05 to 09
10 to 14
15 to 19
20 to 24
25 to 29
30 to 34
35 to 39
40 to 44
45 to 49
50 to 54
55 to 59
60 to 64
65 to 69
70 to 74
75 to 79
80 to 84
85 to 89
90+
age group

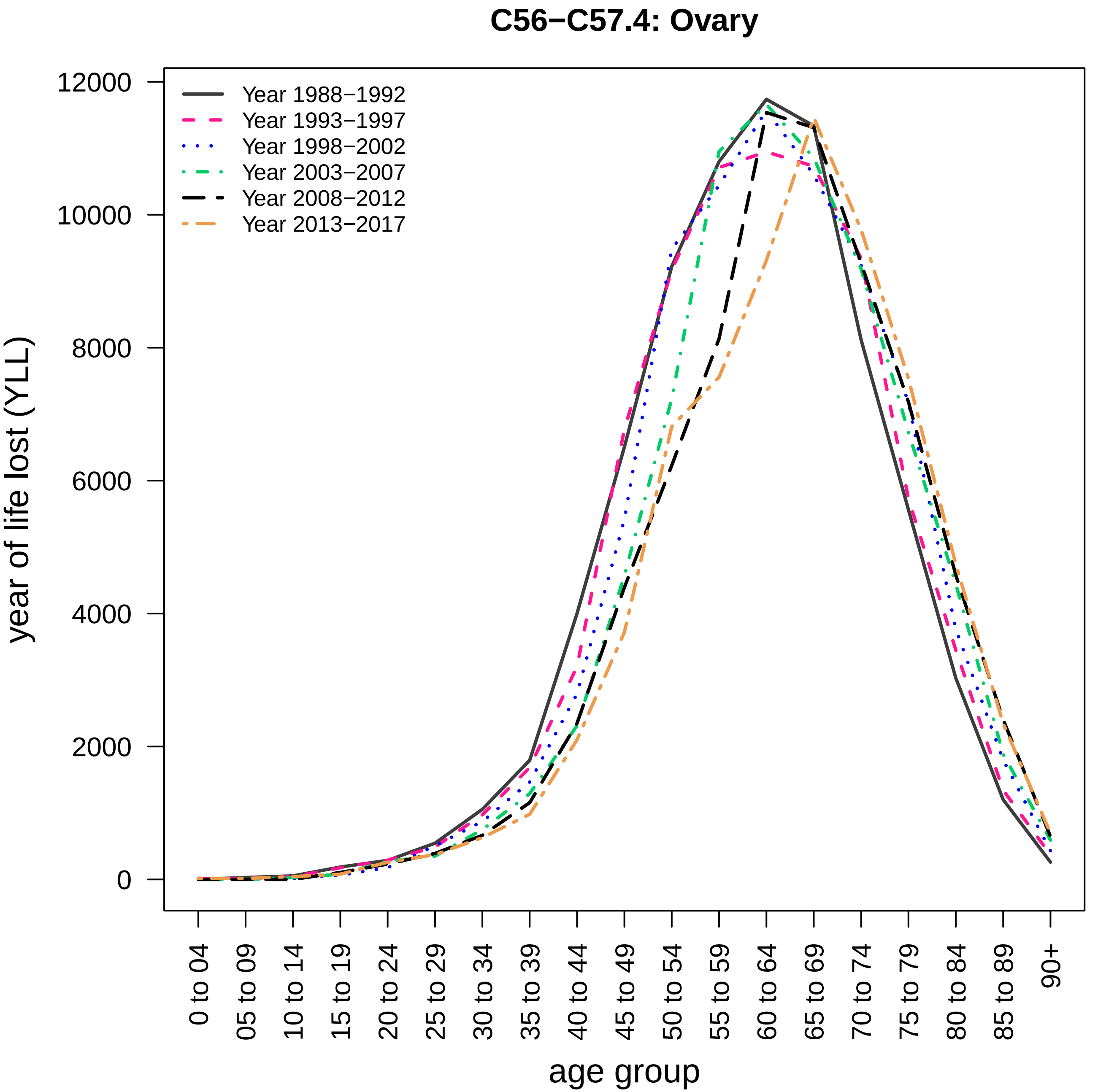

C56−C57.4: Ovary
Year 1988−1992
Year 1993−1997
Year 1998−2002
Year 2003−2007
Year 2008−2012
Year 2013−2017
year of life lost (YLL)
0
2000
4000
6000
8000
10000
12000
0 to 04
05 to 09
10 to 14
15 to 19
20 to 24
25 to 29
30 to 34
35 to 39
40 to 44
45 to 49
50 to 54
55 to 59
60 to 64
65 to 69
70 to 74
75 to 79
80 to 84
85 to 89
90+
age group

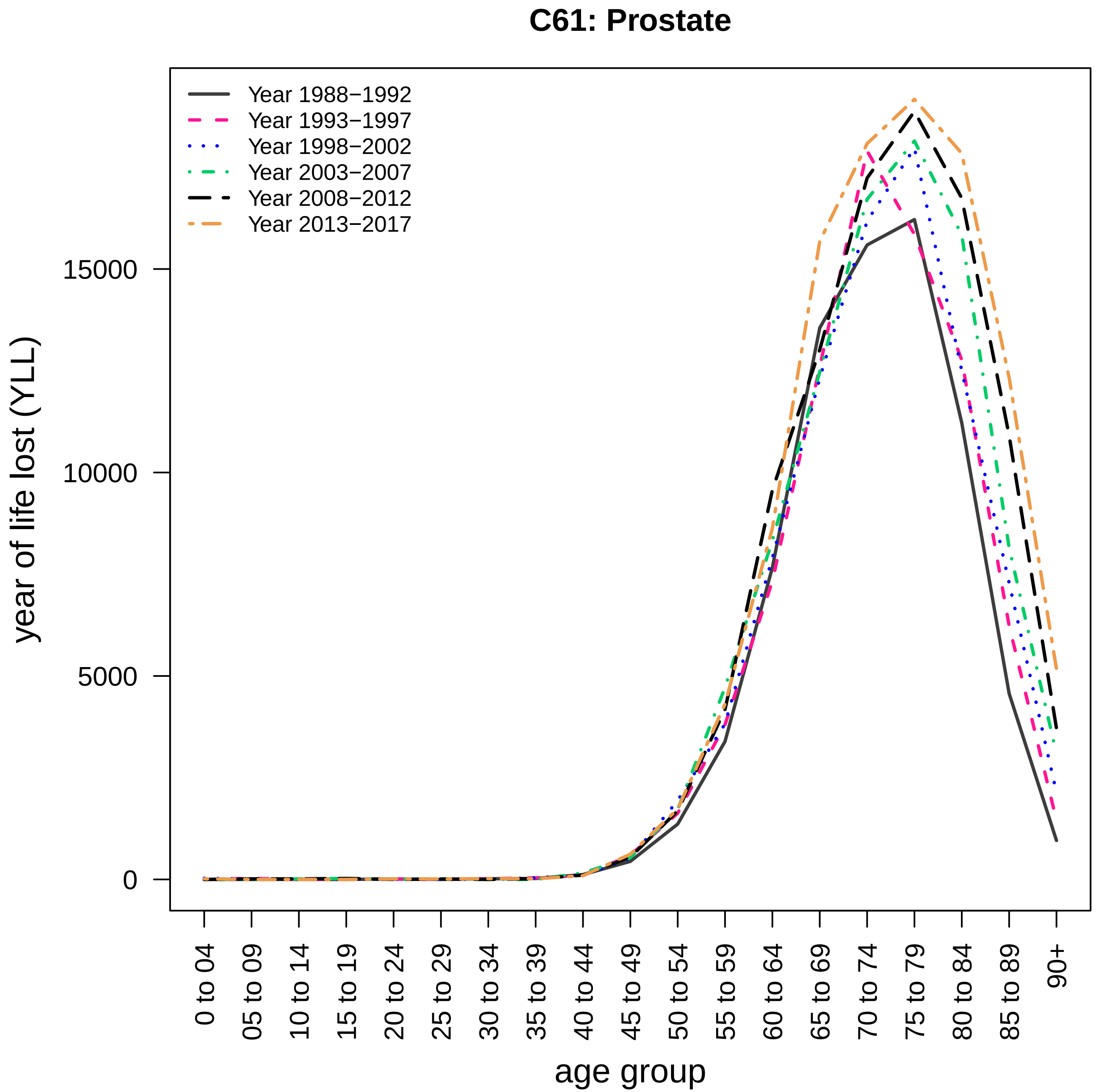
C61: Prostate
Year 1988−1992
Year 1993−1997
Year 1998−2002
Year 2003−2007
Year 2008−2012
Year 2013−2017
year of life lost (YLL)
0
5000
10000
15000
0 to 04
05 to 09
10 to 14
15 to 19
20 to 24
25 to 29
30 to 34
35 to 39
40 to 44
45 to 49
50 to 54
55 to 59
60 to 64
65 to 69
70 to 74
75 to 79
80 to 84
85 to 89
90+
age group

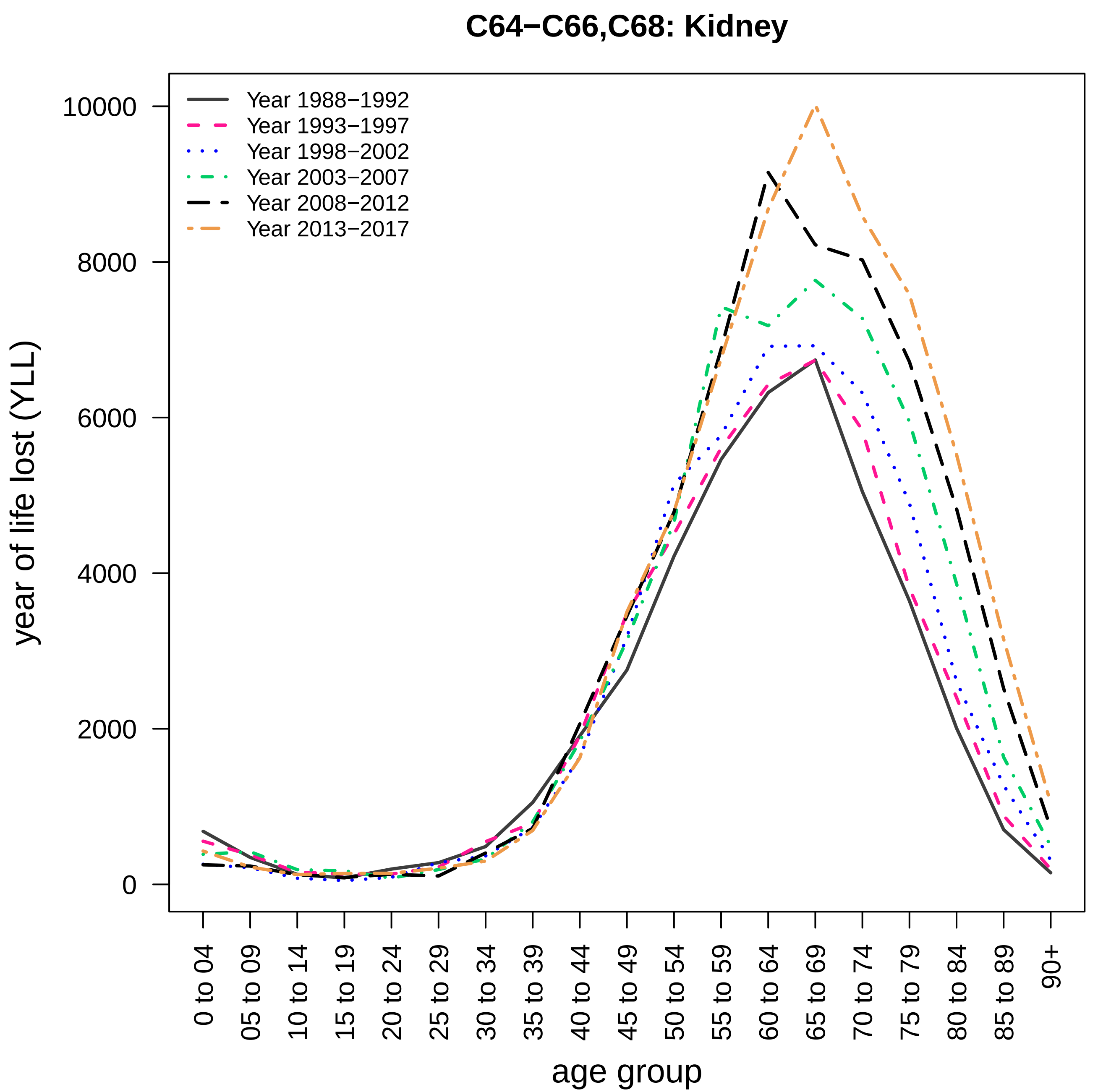

C64−C66,C68: Kidney
Year 1988−1992
Year 1993−1997
Year 1998−2002
Year 2003−2007
Year 2008−2012
Year 2013−2017
year of life lost (YLL)
0
2000
4000
6000
8000
10000
0 to 04
05 to 09
10 to 14
15 to 19
20 to 24
25 to 29
30 to 34
35 to 39
40 to 44
45 to 49
50 to 54
55 to 59
60 to 64
65 to 69
70 to 74
75 to 79
80 to 84
85 to 89
90+
age group

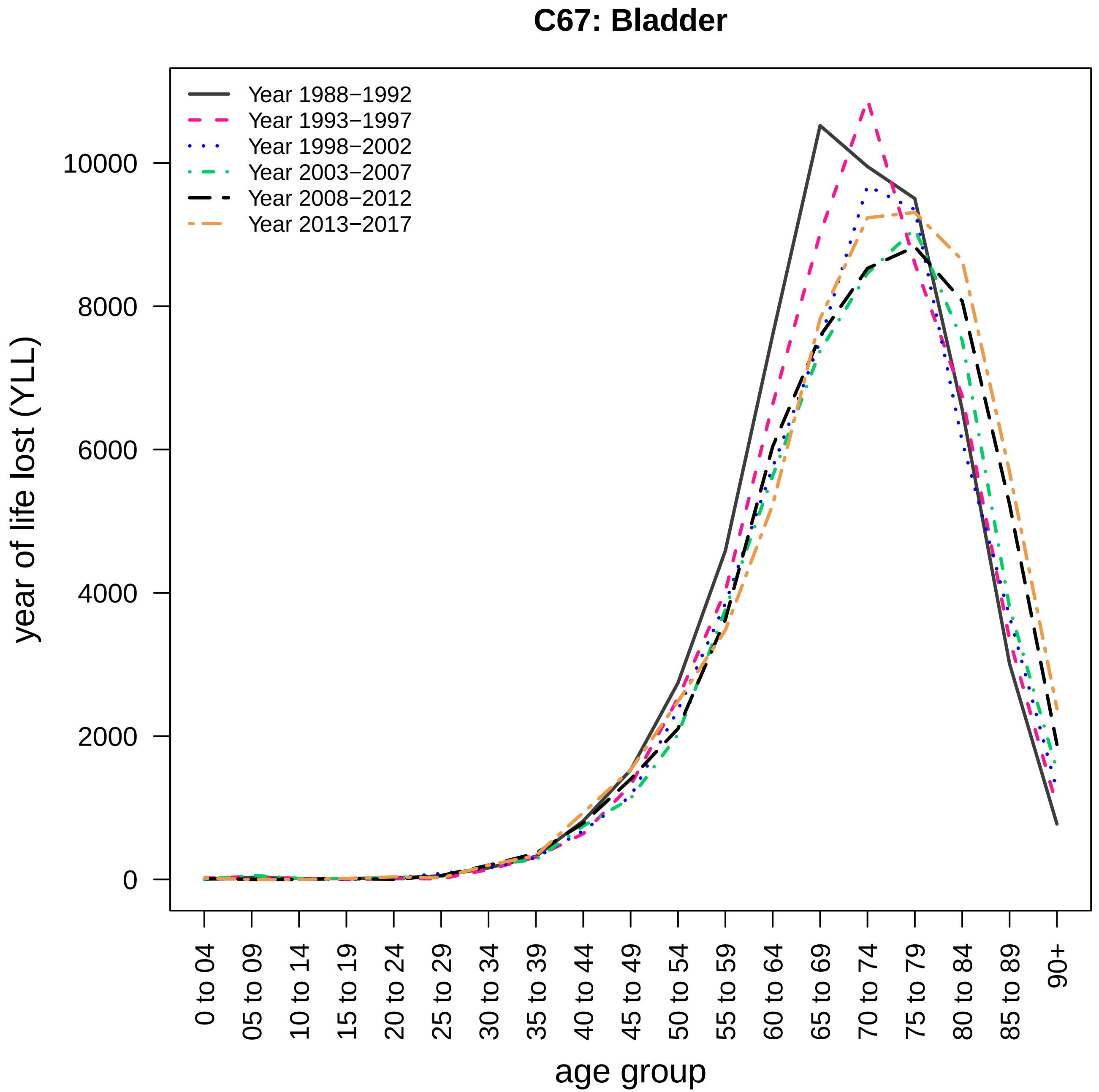

C67: Bladder
Year 1988−1992
Year 1993−1997
Year 1998−2002
Year 2003−2007
Year 2008−2012
Year 2013−2017
year of life lost (YLL)
0
2000
4000
6000
8000
10000
0 to 04
05 to 09
10 to 14
15 to 19
20 to 24
25 to 29
30 to 34
35 to 39
40 to 44
45 to 49
50 to 54
55 to 59
60 to 64
65 to 69
70 to 74
75 to 79
80 to 84
85 to 89
90+
age group

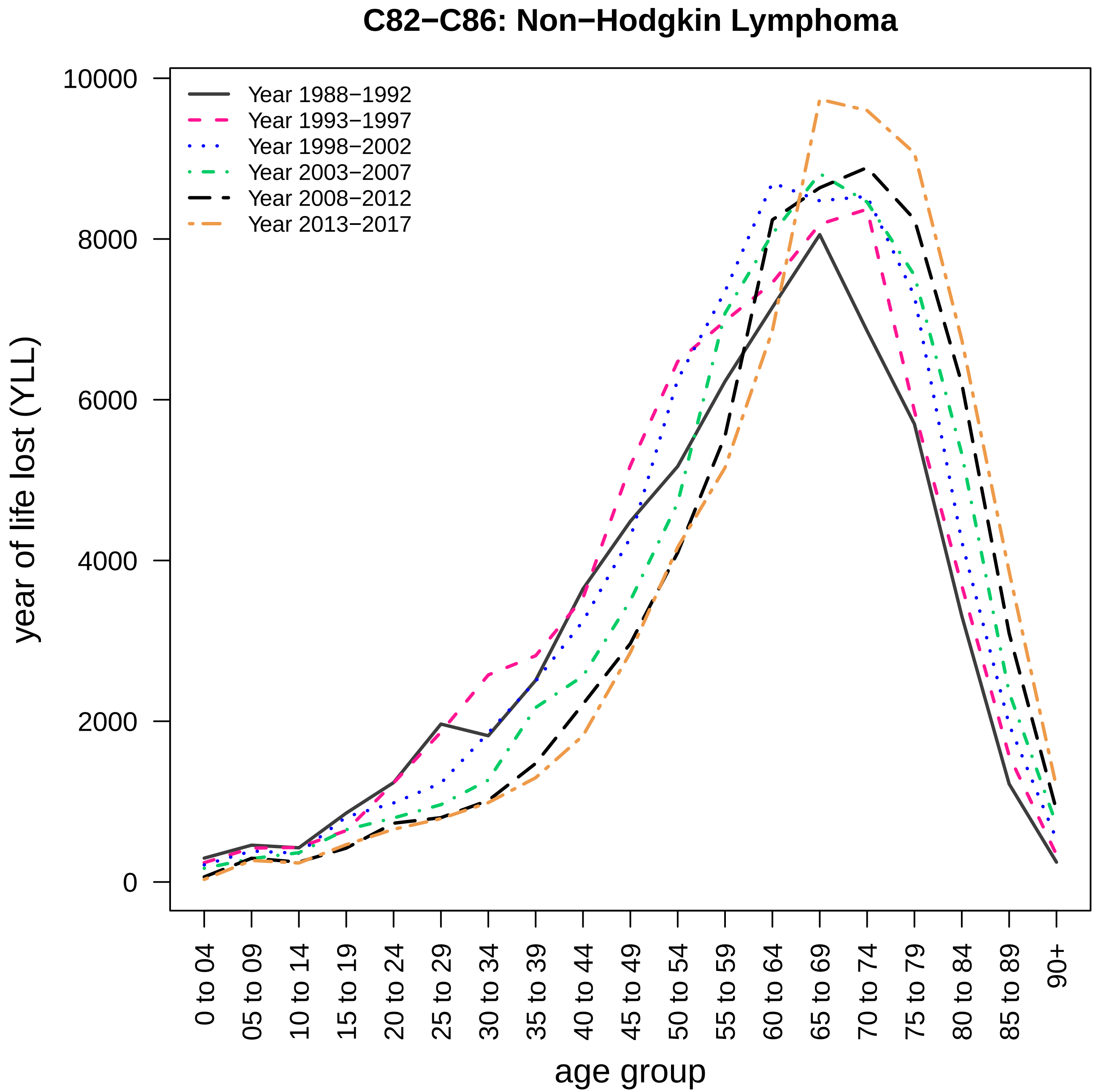
C82−C86: Non−Hodgkin Lymphoma
Year 1988−1992
Year 1993−1997
Year 1998−2002
Year 2003−2007
Year 2008−2012
Year 2013−2017
year of life lost (YLL)
0
2000
4000
6000
8000
10000
0 to 04
05 to 09
10 to 14
15 to 19
20 to 24
25 to 29
30 to 34
35 to 39
40 to 44
45 to 49
50 to 54
55 to 59
60 to 64
65 to 69
70 to 74
75 to 79
80 to 84
85 to 89
90+
age group

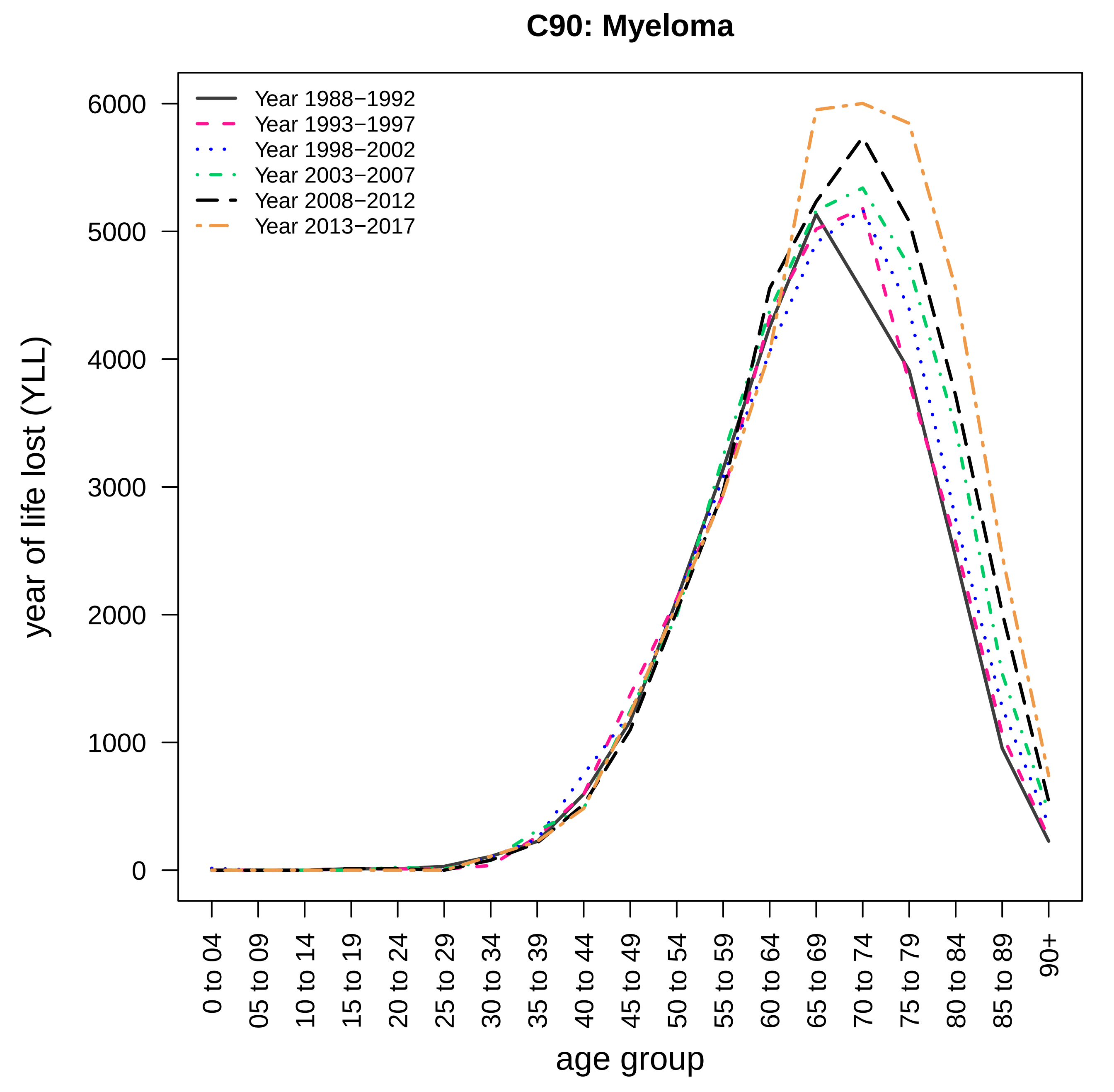

C90: Myeloma
Year 1988−1992
Year 1993−1997
Year 1998−2002
Year 2003−2007
Year 2008−2012
Year 2013−2017
year of life lost (YLL)
0
1000
2000
3000
4000
5000
6000
0 to 04
05 to 09
10 to 14
15 to 19
20 to 24
25 to 29
30 to 34
35 to 39
40 to 44
45 to 49
50 to 54
55 to 59
60 to 64
65 to 69
70 to 74
75 to 79
80 to 84
85 to 89
90+
age group

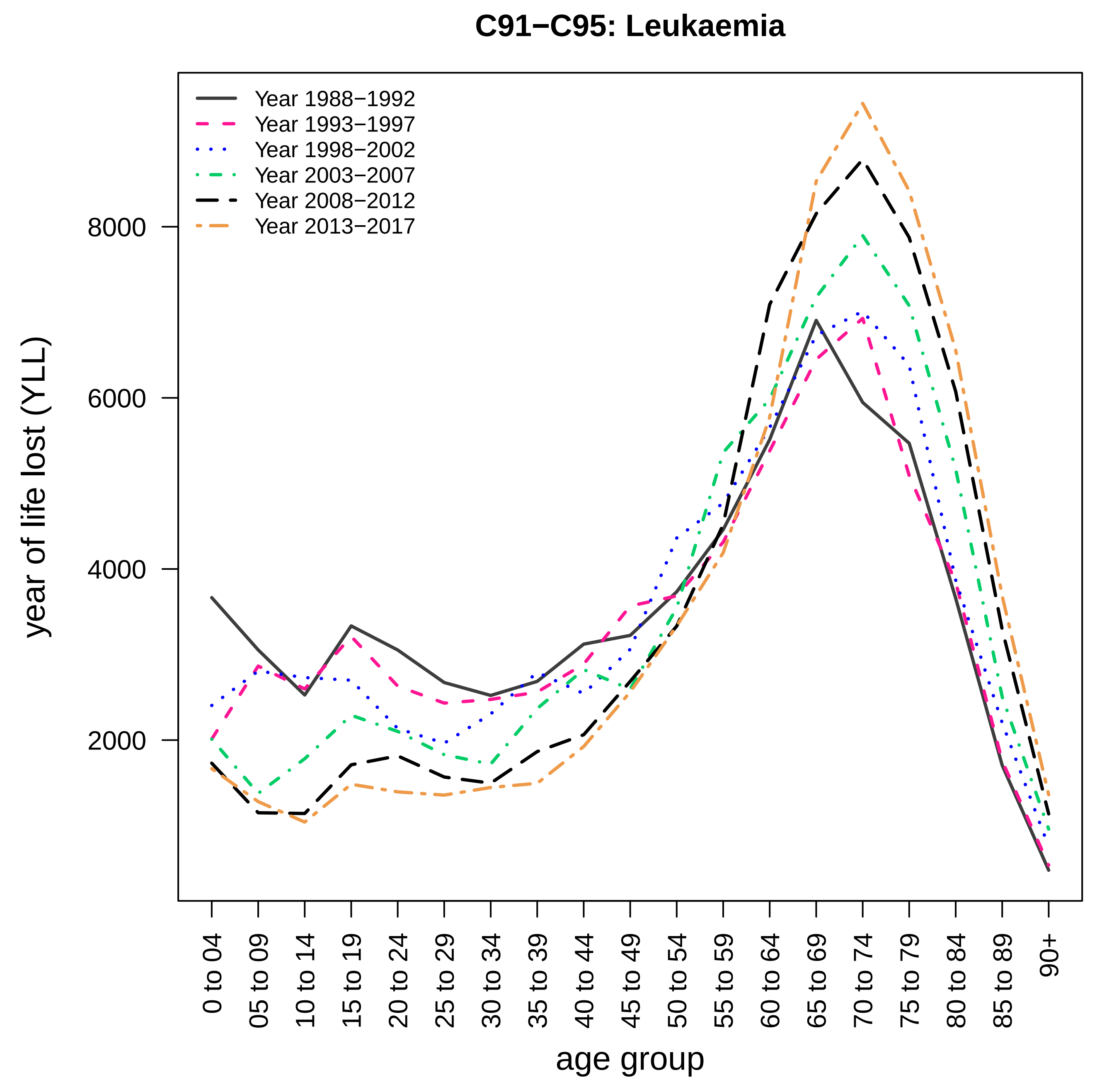
C91−C95: Leukaemia
Year 1988−1992
Year 1993−1997
Year 1998−2002
Year 2003−2007
Year 2008−2012
Year 2013−2017
year of life lost (YLL)
8000
6000
4000
2000
0 to 04
05 to 09
10 to 14
15 to 19
20 to 24
25 to 29
30 to 34
35 to 39
40 to 44
45 to 49
50 to 54
55 to 59
60 to 64
65 to 69
70 to 74
75 to 79
80 to 84
85 to 89
90+
age group
